# Analysis of the complete mitochondrial genome of *Pochonia chlamydosporia* suggests a close relationship to the invertebrate-pathogenic fungi in Hypocreales

**DOI:** 10.1186/s12866-015-0341-8

**Published:** 2015-01-31

**Authors:** Runmao Lin, Chichuan Liu, Baoming Shen, Miao Bai, Jian Ling, Guohua Chen, Zhenchuan Mao, Xinyue Cheng, Bingyan Xie

**Affiliations:** Institute of Vegetables and Flowers, Chinese Academy of Agricultural Sciences, Beijing, 100081 China; College of Plant Protection, Hunan Agricultural University, Changsha, Hunan Province 410128 China; Key Laboratory for Crop Germplasm Innovation and Utilization of Hunan Province, Hunan Agricultural University, Changsha, Hunan Province 410128 China; College of Life Sciences, Beijing Normal University, Beijing, 100875 China

**Keywords:** Nematode egg-parasite fungus, *Pochonia chlamydosporia*, Biological control agent, Mitochondrial genome, *rps3*, Rearrangement, Evolution, Phylogeny

## Abstract

**Background:**

The fungus *Pochonia chlamydosporia* parasitizes nematode eggs and has become one of the most promising biological control agents (BCAs) for plant-parasitic nematodes, which are major agricultural pests that cause tremendous economic losses worldwide. The complete mitochondrial (mt) genome is expected to open new avenues for understanding the phylogenetic relationships and evolution of the invertebrate-pathogenic fungi in Hypocreales.

**Results:**

The complete mitogenome sequence of *P. chlamydosporia* is 25,615 bp in size, containing the 14 typical protein-coding genes, two ribosomal RNA genes, an intronic ORF coding for a putative ribosomal protein (*rps3*) and a set of 23 transfer RNA genes (*trn*) which recognize codons for all amino acids. Sequence similarity studies and syntenic gene analyses show that 87.02% and 58.72% of *P. chlamydosporia* mitogenome sequences match 90.50% of *Metarhizium anisopliae* sequences and 61.33% of *Lecanicillium muscarium* sequences with 92.38% and 86.04% identities, respectively. A phylogenetic tree inferred from 14 mt proteins in Pezizomycotina fungi supports that *P. chlamydosporia* is most closely related to the entomopathogenic fungus *M. anisopliae*. The invertebrate-pathogenic fungi in Hypocreales cluster together and clearly separate from a cluster comprising plant-pathogenic fungi (*Fusarium* spp.) and *Hypocrea jecorina*. A comparison of mitogenome sizes shows that the length of the intergenic regions or the intronic regions is the major size contributor in most of mitogenomes in Sordariomycetes. Evolutionary analysis shows that *rps3* is under positive selection, leading to the display of unique evolutionary characteristics in Hypocreales. Moreover, the variability of *trn* distribution has a clear impact on gene order in mitogenomes. Gene rearrangement analysis shows that operation of transposition drives the rearrangement events in Pezizomycotina, and most events involve in *trn* position changes, but no rearrangement was found in Clavicipitaceae.

**Conclusions:**

We present the complete annotated mitogenome sequence of *P. chlamydosporia*. Based on evolutionary and phylogenetic analyses, we have determined the relationships between the invertebrate-pathogenic fungi in Hypocreales. The invertebrate-pathogenic fungi in Hypocreales referred to in this paper form a monophyletic group sharing a most recent common ancestor. Our *rps3* and *trn* gene order results also establish a foundation for further exploration of the evolutionary trajectory of the fungi in Hypocreales.

**Electronic supplementary material:**

The online version of this article (doi:10.1186/s12866-015-0341-8) contains supplementary material, which is available to authorized users.

## Background

The nematophagous fungus *Pochonia chlamydosporia* (Goddard) Zare & Gams (previously named *Verticillium chlamydosporium*, teleomorph *Metacordyceps chlamydosporia*) is a widespread soil fungus distributed worldwide in nematode suppressive soils. *Pochonia chlamydosporia* can infect the females and eggs of plant endoparasitic nematodes as a facultative parasite [1,2]. It has already demonstrated its efficacy as a BCA against both root-knot nematodes (RKNs; *Meloidogyne* spp.) and cyst nematodes (*Heterodera* spp. and *Globodera* spp.) [2-5], major agricultural pests that cause tremendous economic losses worldwide estimated at 100 billion dollars annually [6].

The fungus *P. chlamydosporia* belongs to the family Clavicipitaceae (Ascomycota: Pezizomycotina: Sordariomycetes: Hypocreales). Based on spore morphology and polymorphisms of the nuclear ribosomal internal transcribed spacer (ITS) region, this species has been recognized as having at least two distinct varieties, *P. chlamydosporia* var. *chlamydosporia* and *P. chlamydosporia* var. *catenulate* [7,8]. Many members of Clavicipitaceae are invertebrate-pathogenic fungi. It was previously suggested that the host relatedness and host habitat hypotheses could explain the evolution of parasite-host relationships [9]. Therefore, exploring the phylogenetic relationships and evolutionary trajectories of the invertebrate-pathogenic fungi in Clavicipitaceae is a topic of interest. Multi-gene phylogenetic analyses showed that Clavicipitaceae is paraphyletic and consists of three well-defined clades, at least one of which is shared with the members of another fungal family (Hypocreaceae) [10]. The phylogenetic placement of the genus *Pochonia* in the family Clavicipitaceae was inferred from six nuclear genes (nrSSU, nrLSU, beta-tubulin, EF-1alpha, *RPB1*, *RPB2*) and one mt gene (*atp6*) using phylogenetic analysis, and the phylogenetic trees inferred from different genes produced different topological structures [10]. Therefore, a comparison of larger scale genome sequences is necessary because it can provide deep insights into the phylogenetic relationships and evolution of the fungi in Clavicipitaceae. Recently, the complete nuclear genome sequence of *P. chlamydosporia* was published. Based on a phylogenetic analysis inferred from nuclear genome sequences, *P. chlamydosporia* appeared to be most closely related to the *Metarhizium* species [11].

Mitochondrial markers can be successfully applied in evolutionary biology and systematics because mt genomes often evolve faster than nuclear genomes and allow for robust phylogenetic analyses based on the conserved proteins of the oxidative phosphorylation system [12]. The continuously increasing number of recent fungal mt genome studies – more than 165 complete fungal mt genomes are available today at NCBI - provide powerful tools for comparative studies to reveal the patterns and mechanisms of mitogenome evolution [13,14]. Thus far, fungal mitogenomes vary considerably in size, the largest being 147,264 bp long in *Rhizoctonia solani* AG1 IA (Basidiomycota) [15] and the smallest being 18,844 bp long in *Hanseniaspora uvarum* (Ascomycota) [16]. Researchers have demonstrated that different lengths of intronic sequences and intergenic regions result in different sizes of mitogenomes [17-19], particularly for the mitogenome of *Moniliophthora perniciosa* in Basidiomycota, which has a length of 109,103 bp and encodes several hypothetical ORFs in the larger sized intergenic regions and introns [19]. However, a depiction of the size variation and related mechanisms in Ascomycota has not yet been reported. Moreover, the nearly ubiquitous presence of *rps3* genes, which are encoded in the group I introns within *rnl* genes, has been attributed to a vertical mode of inheritance rather than horizontal inheritance during evolution [20]. In one study, the molecular evolution of the *rps3* gene in filamentous Ascomycetes fungi (especially Ophiostomatoid fungi) was analysed, and the group I intron-encoded version of *rps3* appeared to have a rather complex evolutionary history in ascomycetes fungi [21]. However, only a few fungi in Hypocreales were included in that study. To better understand the evolution of the fungal mitogenome in Hypocreales, a phylogenetic analysis of the *rps3* genes is required. Furthermore, it was reported that *trn* genes are able to change their location in genomes and participate in horizontal gene transfer (HGT) events, as they have editing, excision and integration capabilities [22,23]. Because changes in *trn* location are relatively rare events, the locations of *trn* genes in fungal mt genomes have been used to study fungal evolution and phylogenetic signals [22]. To date, 20 different types of aminoacyl-tRNAs (aa-tRNAs) have been identified in fungal mitogenomes, and conserved *trn* clusters are found in Pezizomycotina by employing comparative genomic approaches [12,24-26]. The distribution of *trn* genes may contribute to gene order variation in fungal mitogenomes [22]. Six operations have been used to explain rearrangements in mt gene order: inversion, transposition, reverse transposition, tandem-duplication-random-loss [27], deletion and replication slippage [28]. However, the evolution of gene orders in Pezizomycotina mitogenomes is suggested to be mainly driven by transpositions [12]. As in animal mt genomes, *trn* in fungal mitogenomes might have played a role in gene shuffling as they intervene between genes and can act like mobile elements [29,30]. Taking advantage of published fungal mitogenomes and the novel *P. chlamydosporia* mitogenome, we investigated the *trn* rearrangements in Pezizomycotina, and addressed the role of rearrangements in evolution.

In this paper, the *P. chlamydosporia* strain 170 isolated from a RKN in China was sequenced using Illumina sequencing, and a complete mt genome of 25,615 bp was obtained. We aim to (i) present a complete and annotated mt genome sequence of *P. chlamydosporia*, (ii) compare the mt genome of *P. chlamydosporia* with the genomes of other fungi in Pezizomycotina to identify the common and specific characteristics of the mt genomes of invertebrate-pathogenic fungi, and (iii) provide powerful insights into the evolution and phylogenetic relationships of the invertebrate-pathogenic fungi in Hypocreales.

## Results

### General characteristics of the mt genome in *P. chlamydosporia*

**Figure 1:**
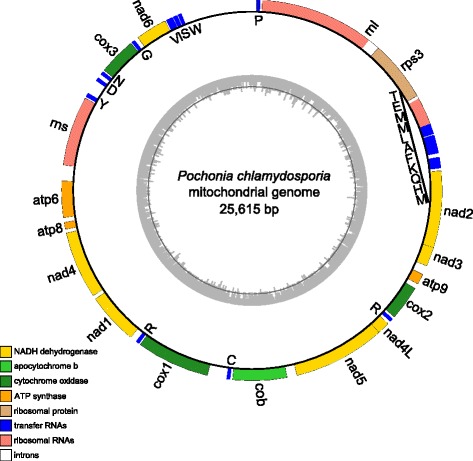
**Functional map of the complete mitogenome of** ***P. chlamydosporia*** **.** All genes are transcribed on the same strand. There are 15 encoding genes, 23 transfer RNA genes and 2 ribosomal RNA genes (*rns* and *rnl*). The circular map was generated using OrganellarGenomeDRAW [31].

The complete mt genome of *P. chlamydosporia* is a circular DNA molecule with a length of 25,615 bp. The low G + C content (28.3%) is similar to other fungal mitogenomes in Clavicipitaceae (Table 1). The mitogenome encodes an essential set of conserved genes including three cytochrome c oxidase subunits (*cox1*, *cox2*, *cox3*), apocytochrome b (*cob*), three ATP synthase subunits (*atp6*, *atp8*, *atp9*), seven subunits of NADH dehydrogenase (*nad1*, *nad2*, *nad3*, *nad4*, *nad4L*, *nad5*, *nad6*), the small and large ribosomal RNA subunits (*rns*, *rnl*), an intronic ORF coding for a putative *rps3*, and 23 *trn*. All of the 40 genes are encoded on the same DNA strand (Figure 1). The set of *trn* genes found (including two *trnA* genes and three *trnM* genes) is the smallest set currently known for Pezizomycotina fungi (Table 1), but the *trn* genes can recognize the codons for all 20 amino acids. In total, the coding regions (CRs), of which the 14 typical protein-coding genes related to oxidative phosphorylation account for 13,068 bp, and together with the *rps3* (1,317 bp) within the *rnl* group I intron (1,652 bp, in total), cover 56.16% of the total mt genome, and the rest are *trn*, rRNA genes and intergenic regions.

**Table 1:** **General features of the mitogenomes**

**Species**	**Length (bp)**	**GC (%)**	**Coding genes**	***trn***	**rRNAs**	**Accession**
*Pochonia chlamydosporia*	25,615	28.3	15	23	2	KF479445
*Metarhizium anisopliae*	24,673	28.4	15	24	2	AY884128
*Beauveria bassiana*	29,961	27.2	15	25	2	EU371503
*Beauveria pseudobassiana*	28,006	27.5	15	25	2	NC_022708
*Cordyceps brongniartii*	33,926	27.3	15	25	2	NC_011194
*Lecanicillium muscarium*	24,499	27.1	15	25	2	AF487277
*Cordyceps militaris*	33,277	26.8	15	26	2	NC_022834
*Hypocrea jecorina*	42,130	27.2	19	26	2	AF447590
*Fusarium solani*	62,978	28.9	30	25	2	NC_016680
*Fusarium graminearum*	95,676	31.8	50	28	2	NC_009493
*Fusarium verticillioides*	53,735	32.6	21	27	2	NC_016687
*Fusarium oxysporum*	34,477	31.0	16	25	2	AY945289
*Verticillium dahliae*	27,184	27.3	15	25	2	DQ351941
*Neurospora crassa*	64,840	36.1	28	28	2	Broad Institute
*Aspergillus fumigatus*	31,762	25.4	20	31	2	JQ346809
*Aspergillus niger*	31,103	26.9	16	25	2	NC_007445
*Aspergillus tubingensis*	33,656	26.8	16	25	2	NC_007597
*Trichophyton rubrum*	26,985	23.5	17	25	2	NC_012824
*Epidermophyton floccosum*	30,910	23.4	24	25	2	NC_007394
*Paracoccidioides brasiliensis*	71,335	21.1	17	25	2	NC_007935
*Candida parapsilosis*	32,745	23.8	20	24	2	NC_005253

Using the abundant sequenced resources available (the average coverage per base is 1,058 ×, see the Methods section), the sequence variance of the *P. chlamydosporia* mitogenome was investigated, but no single nucleotide polymorphism (SNP) was found. This indicates that a high quality mitogenome was assembled. Moreover, we compared the published mt gene sequences of *P. chlamydosporia* with our genome sequence. In total, 12 mt gene sequences from NCBI are available, including six coding segments in the IMI 113169 strain (*nad3-atp9*, *atp6*, *rns*, *nad1*, *nad3*, *cox3*), four genes in the IMI 156157 strain (*rns*, *nad1*, *nad3*, *cox3*), one gene in the CBS 101244 strain (*atp6*) and one gene in the CBS 504.66 strain (*atp6*). Our analysis showed that 11 sequences were matched to our genome sequence with identities greater than or equal to 95% (Additional file 1: Table S1), suggesting that these gene sequences are highly conserved in *P. chlamydosporia* strains.

### Phylogenetic relationships of *P. chlamydosporia* to other fungi in Pezizomycotina

**Figure 2:**
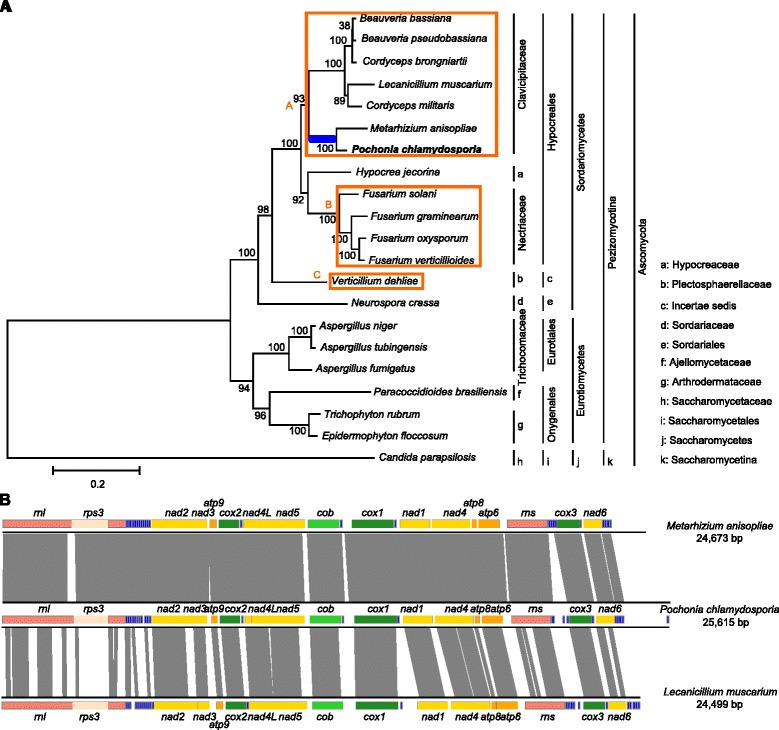
**The mitogenome phylogenetic analysis and comparative analysis. A)**. Phylogenetic placement of *P. chlamydosporia* using 21 fungal mitogenomes. The phylogeny was generated based on 14 conserved encoded proteins (*cox1-3*, *cob*, *nad1-6*, *nad4L*, *atp6*, *atp8-9*) using a ML approach. *C. parapsilosis* was used as an outgroup. *P. chlamydosporia* is closely related to *M. anisopliae* with a bootstrap support value of 100%. **B)**. Synteny comparisons between *P. chlamydosporia* and *M. anisopliae*, *P. chlamydosporia* and *L. muscarium*. Syntenic regions were identified by performing BLASTN with a threshold of 1e-5, and alignment blocks of less than 100 bp are not shown.

Utilizing published fungal mitogenomes in addition to the novel *P. chlamydosporia* mitogenome, the phylogenetic relationships in 20 species in Pezizomycotina were inferred, using *Candida parapsilosis* as an outgroup (Figure 2A). Phylogenetic trees were constructed based on the 14 conserved protein-coding genes associated with the oxidative phosphorylation system (*cox1-3*, *cob*, *atp6*, *atp8-9*, *nad1-6* and *nad4L*) using a Maximum Likelihood (ML) approach. The topological structures based on both nucleotide and amino acid sequences were identical, and a clear genealogical relationship was shown (Figure 2A). *P. chlamydosporia* is most closely related to the entomopathogenic fungus *M. anisopliae*, with a bootstrap value of 100%, which is similar to the result of phylogenetic analysis based on genome-encoded orthologous proteins [11]. The two fungi form a cluster with five additional entomopathogenic fungi, namely, *Cordyceps brongniartii*, *Beauveria pseudobassiana*, *B. bassiana*, *Lecanicillium muscarium* and *C. militaris*. Interestingly, although both *P. chlamydosporia* and *L. muscarium* were once put into the same genus (*Verticillium*), the two species are assigned to different sub-clades in the phylogenetic tree. However, all of the invertebrate-pathogenic fungi cluster into a clade (clade A) clearly separated from the plant-parasitic fungi, including four *Fusarium* plant pathogens (*F. solani*, *F. graminearum*, *F. oxysporum* and *F. verticillioides*), which form clade B with the fungus *Hypocrea jecorina* that specializes in colonizing pre-degraded wood. Both invertebrate-pathogenic and plant-pathogenic fungi shared a common ancestor in clade C represented by a soil born plant pathogen, *Verticillium dahliae*, suggesting that Hypocreales is a monophyletic group (Figure 2A). The phylogeny shows that plant pathogenic and invertebrate-pathogenic fungi in Hypocreales form independent clusters and have evolved separately. A third group comprises fungi that infect animals or humans, including several members of Eurotiomycetes, i.e., *Aspergillus fumigates*, *Paracoccidioides brasiliensis*, *Epidermophyton floccosum* and *Trichophyton rubrum*; this group is distant from *P. chlamydosporia* and the entomopathogenic fungi. The phylogenetic relationships inferred from mt proteins are principally consistent with the current Pezizomycotina taxonomic system (Figure 2A).

### Comparison of mitogenome organization in *P. chlamydosporia* and related species

We first compared the mitogenomes of *P. chlamydosporia*, *M. anisopliae* and *L. muscarium* (previously widely known as *Verticillium lecanii*), and found that they are highly similar in both size (25,615 bp, 24,673 bp and 24,499 bp, respectively) and G + C content (28.3%, 28.4% and 27.1%, respectively) (Table 1). The total length of the gene regions (including protein-coding genes, *trn* and rRNA genes) in *P. chlamydosporia* mt genome (20,997 bp) is 142 bp less than that of *L. muscarium* and 208 bp less than that of *M. anisopliae*. However, the entire length of the *P. chlamydosporia* mitogenome is 1,116 bp and 942 bp larger than *L. muscarium* and *M. anisopliae* respectively, mainly due to longer intergenic regions in *P. chlamydosporia*. We also compared the contents of the simple repeat motifs in each of the genomes, including low complexity sequences (such as AT-rich sequences), simple repeats (such as (TAA) n), inverted repeats and palindromes, which have been previously suggested to be putative elements for recombination or regulation [24]. It was found that ~4.14% (1,061 bp, including 948 bp tandem repeats, 64 bp inverted repeats and 172 bp palindromes with 123 bp overlaps between tandem repeats and palindromes) of the *P. chlamydosporia* mitogenome consists of repeat sequences compared with only ~2.71% (664 bp) of the *L. muscarium* mitogenome and ~3.99% (985 bp) of the *M. anisopliae* mitogenome. The distributions of repeat elements are also different (Additional file 1: Figure S1). In *L. muscarium*, there are no repeat sequences located in the regions between *nad6* and *rnl*, *nad5* and *cob*, *rns* and *cox3*. However, in *M. anisopliae* and *P. chlamydosporia*, there are no repeat sequences in the regions between *rnl* and *nad2*, *cox3* and *nad6*. There are repeat sequences residing in the regions between *rns* and *cox3* in *P. chlamydosporia*, but only one AT rich motif is found in this region in *M. anisopliae* (Additional file 1: Figure S1). Repeat sequences in this region display the unique mitogenomic features of *P. chlamydosporia*.

In addition, sequence similarity studies and syntenic gene analyses were performed comparing *P. chlamydosporia* with five other mitogenomes, including *M. anisopliae*, *L. muscarium*, *B. bassiana*, *V. dahliae* and *A. fumigatus*. The analyses show identical gene order in *M. anisopliae*, *L. muscarium*, *B. bassiana* and *P. chlamydosporia* mt genomes. *V. dahliae*, as shown in previous work [32], displayed differences at the regions *nad6*-*rnl*, *nad3*-*atp9* and *cox1*-*nad1* with a gene order of *nad6*-*atp9*, *nad3*-*nad1* and *cox1*-*rnl* instead (Additional file 1: Figure S2). A larger difference of gene order is observed between the mitogenomes of *P. chlamydosporia* and *A. fumigatus* (Additional file 1: Figure S2). Comparing the two mitogenomes of *P. chlamydosporia* and *M. anisopliae*, 22,331 bp (90.50%) of *M. anisopliae* mitogenome sequences match to 22,290 bp (87.02%) of *P. chlamydosporia* mitogenome sequences with 92.38% identity determined by BLASTN (Figure 2B). A long-range synteny between the *P. chlamydosporia* and *M. anisopliae* mt genomes is observed. The majority of the unmatched sequences reside within the intergenic regions between *nad6* and *rnl*. However, comparing the *P. chlamydosporia* and *L. muscarium* mitogenomes, 15,026 bp (61.33%) of the *L. muscarium* mitogenome sequences match to 58.72% of the *P. chlamydosporia* mitogenome sequences with 86.04% identity (Figure 2B). Comparisons of *P. chlamydosporia* mt genomes with the other three species (*B. bassiana* and *V. dahliae* in Sordariomycetes, and *A. fumigatus* in Eurotiomycetes) showed that only 15,624 bp (52.15%) of *B. bassiana*, 13,412 bp (49.34%) of *V. dahliae* and 8,147 bp (25.65%) of *A. fumigatus* mitogenomes matched to *P. chlamydosporia* mt sequences with 87.52%, 84.62% and 82.82% identities, respectively (Additional file 1: Figure S2). Although the sequences of most of the mt genes in *V. dahliae* matched to homologous genes in *P. chlamydosporia* mitogenome, the alignments of some genes (such as *rns*, *nad5* and *nad6*) between the two species present multiple blocks, and no alignments are found in the intergenic regions in the *P. chlamydosporia* and *V. dahliae* mitogenomes. Based on the above syntenic analysis results, an evolutionary relationship between *P. chlamydosporia* and the other fungi is also indicated, that is, that *P. chlamydosporia* is most closely related to *M. anisopliae*. The result is similar to that obtained from the genomic DNA analysis [11].

### Analysis of mitogenome size variation among the fungi in Sordariomycetes

**Figure 3:**
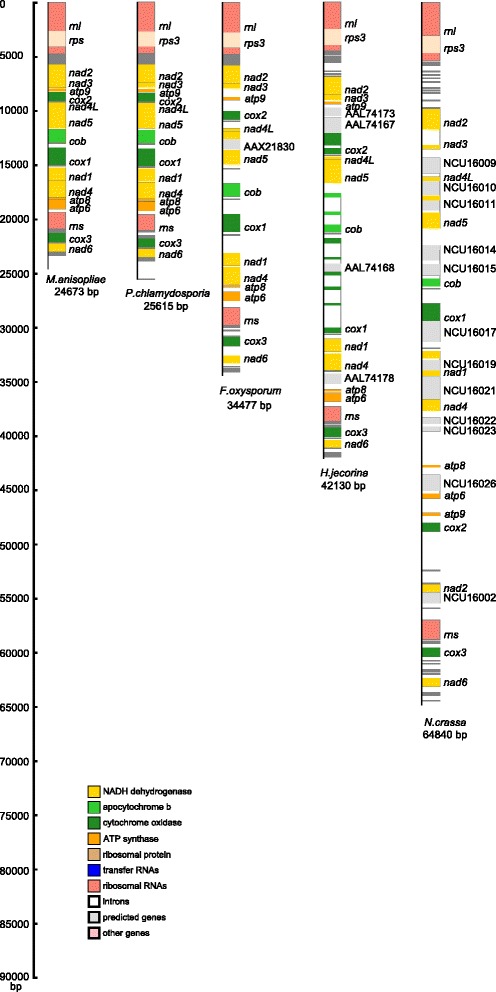
**A comparison of the mitogenomes of five Pezizomycotina fungi with considerable size differences.** There are 15, 15, 16, 19, and 28 protein-coding genes in *P. chlamydosporia*, *M. anisopliae*, *F. oxysporum*, *H. jecorina*, and *N. crassa*, respectively. Size comparisons indicate that the larger the mitogenomes are primarily due to larger intergenic regions, secondarily to introns, and to a lesser extent to longer coding regions.

The sizes of the mitogenome in the fungi in Sordariomycetes vary significantly, from 24,673 bp in *M. anisopliae* to 95,676 bp in *F. graminearum* (Table 1). To identify the causes of this mitogenome size variation in Sordariomycetes, five mitogenomes (*P. chlamydosporia*, *M. anisopliae*, *F. oxysporum*, *H. jecorina* and *Neurospora crassa*) of different sizes were used for a comparative analysis (Figure 3). Despite the size variance ranging from 24,673 bp in *M. anisopliae* to 64,840 bp in *N. crassa*, 15 common mt protein-coding gene sets, including *cox1-3*, *cob*, *nad1-6*, *nad4L*, *atp6*, *atp8-9* and *rsp3*, are encoded in all of the five genomes. We calculated the size of the CRs, *trn* regions, rRNA regions, introns and intergenic regions in each mitogenome separately (Additional file 1: Table S2). In *P. chlamydosporia* mitogenome, the lengths of the CRs, *trn* regions, rRNA regions, introns and intergenic regions are 14,385 bp (56.16%), 1,697 bp (6.63%), 6,232 bp (24.33%), 335 bp (1.31%) and 4,618 bp (18.03%) of the genome sequences, respectively. A comparison of the five mitogenomes showed that, in general, the length of each region (CRs, *trn* regions, rRNA regions, introns and intergenic regions) increases with genome size, as well as the proportions of introns and intergenic regions, with the exception of *H. jecorina*, which has the smallest length of rRNA genes but the largest length of introns of the five mitogenomes. In addition, the length of its intergenic regions is less than that of *F. oxysporum*, although the genome size of *H. jecorina* is larger than that of *F. oxysporum* (Additional file 1: Table S2). The results indicate that, in Sordariomycetes, CRs, intergenic regions and introns are the three main contributors to mitogenome size variation, with the length of intergenic regions or introns being the primary contributor in most of the mitogenomes. The length of the CRs in *N. crassa* and *H. jecorina* is the second highest contributor because several intron-encoded ORFs and unidentified reading frames exist in their intergenic regions (Additional file 1: Table S3).

### Codon usage in the fungal mitogenomes of Pezizomycotina

We analysed codon usage bias in the mitogenome of *P. chlamydosporia*, and compared it with other fungal mitogenomes in Pezizomycotina. Genetic code four [33] was used to transcribe the coding gene sequences in the *P. chlamydosporia* mt genome. A total of 58 codons were found, including the high-frequency codons TTA (Leu), ATA (Ile), GTA (Val) and TTC (Phe) (Additional file 1: Table S4), which are amino acids with hydrophobic side chains. Six codons (CTC, CTG, AGG, CGC, CGG and TGG) were absent in *P. chlamydosporia* but present in the closely related species *M. anisopliae*. However, two codons (CGA, TAG) were absent in *M. anisopliae* but present in *P. chlamydosporia*. Clearly, nearly in all of the fungal mitogenomes, codon usage is biased strongly towards codons ending in A or T, and more than 84% of the codons in *P. chlamydosporia* mitogenomes end in A or T (Additional file 1: Table S5), possibly due to the high AT content found in fungal mitogenomes. Similar results were obtained from *M. anisopliae* and *L. muscarium* mitogenomes [24,30].

### Molecular evolution of *rps3* in Pezizomycotina

We identified and annotated the *rps3* genes in the 20 fungal mitogenomes. The length of the *rps3* sequences ranged from 1,140 bp in the *T. rubrum* and *E. floccosum* mitogenomes to 1,545 bp in the *F. verticillioides* mitogenome (Additional file 1: Table S6). When the 20 Rps proteins were aligned by BLASTP the similarity levels were greater than or equal to 30%. However, the C-terminal domains of these proteins obtained from the Pfam database indicated a common function (Additional file 1: Figure S3A). The addition of 19 more fungal *rps3* gene sequences showed matches with E-value cutoffs of less than 2e-29, hence they were omitted from comparisons. E-value cutoffs of 0 were obtained for the *rps3* genes of *F. oxysporum*, *M. anisopliae*, *H. jecorina*, *F. solani*, *F. graminearum* and *F. verticillioides* (Additional file 1: Figure S3B). The unique evolutionary relationships among these species can be elucidated from phylogenetic analysis based on the sequences of the *rps3* genes (Additional file 1: Figure S3C). A phylogenetic tree shows that *P. chlamydosporia rps3* is more closely related to *F. oxysporum* and *M. anisopliae rps3*. They then group together with *H. jecorina* and other three *Fusarium* species to form clade A. Clade A separates distinctly from clade B, which contains the other five entomopathogenic fungi (*L. muscarium*, *C. militaris*, *B. pseudobassiana*, *C. brongniartii* and *B. bassiana*) (Additional file 1: Figure S3C). Evidently, the phylogenetic relationships among the species of Hypocreales inferred from the *rps3* genes are different from those inferred from the other 14 protein-coding genes (Figure 2A).

To measure the rate and selective pressure of the *rps3* genes, we calculated nonsynonymous/synonymous substitution rate ratios (ω = d_N_/d_S_) by adopting different models (see the Methods section). The dN/dS value for the 20 *rps3* genes is 0.090 using the CODEML program with a JTT model in PAML [34]. Because every functional protein contains amino acid sites under selective constraints, averaging the evolutionary rates across sites leads to a lower capacity to detect positive selection. Thus, we adopted the site models M1 (neutral), M2 (selection), M7 (beta) and M8 (beta & ω) for the ω ratio analysis [35]. Because the M7-M8 comparison is a very stringent test of positive selection [36], we calculated the value of the likelihood ratio test (LRT) statistic (2Δ = 25.854, P-value = 2.432e-06, the Chi-square test with degrees of freedom, i.e. d.f. = 2), and the significant results of this comparison clearly indicates signals of positive selection in the *rps3* genes. To further confirm whether the Hypocreales *rps3* genes are evolving under positive selection or not, we took the *rps3* sequence of *T. rubrum* as a reference to calculate the dN and dS of each *rps3* sequence with the reference sequence using DnaSP [37] and CODEML [34]. Similar results were obtained, that is, dN/dS > 1 in Hypocreales (Additional file 1: Figure S3C and Table S7). The above results indicate that the fungal *rps3* protein is not only under functional constraints but also under positive selection in Hypocreales.

### *trn* gene distribution and putative gene rearrangements in Pezizomycotina

**Figure 4:**
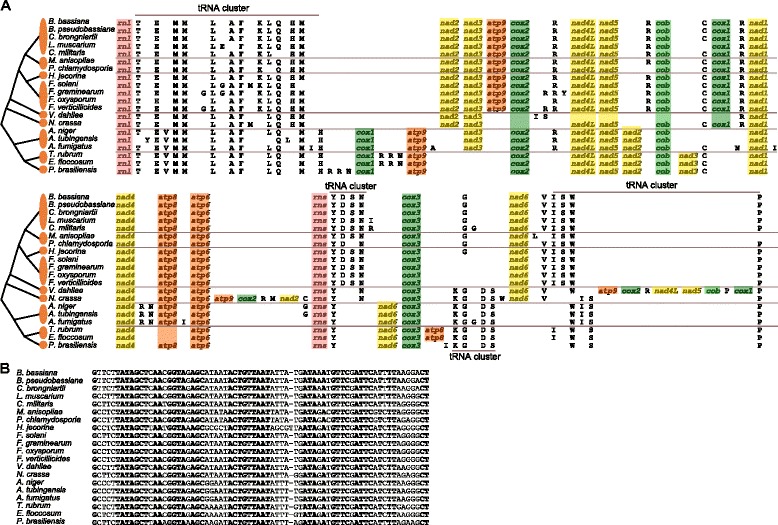
**The schematic representation of** ***trn*** **clusters. A)**. Analysis of the *trn* genes residing in the complete mitogenomes of 20 fungi that belong to nine families. The nine families are Clavicipitaceae, Hypocreaceae, Nectriaceae, Plectosphaerellaceae, Sordariaceae, Lasiosphaeriaceae, Trichocomaceae, Ajellomycetaceae and Arthrodermataceae. **B)**. An example of high sequence similarities in *trn* genes (*trnN*). The consensus bases are marked in boldface.

The distribution of *trn* genes on mt genomes is considered as a factor that potentially contributes to the gene order variation in Pezizomycotina [22]. Firstly, we analyzed the distribution of 23 *trn* genes in the *P. chlamydosporia* mitogenome and found that they cluster into three groups (YDN, VISWP and TEMMLAFKQHM), with the exception of four *trn* genes (*trnG*, *trnR*, *trnC*, *trnR*) that scatter as a single gene throughout the mt genome (Figure 1). Then, we compared the 13 *trn* genes (*trnD*, *trnE*, *trnF*, *trnH*, *trnI*, *trnK*, *trnL*, *trnM*, *trnN*, *trnR*, *trnS*, *trnW*, and *trnY*) in the 20 fungal mitogenomes of Pezizomycotina and found that they are presented in all mitogenomes whereas some of the other seven *trn* genes (*trnA*, *trnC*, *trnG*, *trnP*, *trnQ*, *trnT* and *trnV*) are often missing from a fungal mitogenome (Figure 4A) as reported in previous studies [24,25,30,32,38,39]. Some *trn* genes have multiple copies, such as *trnM*, which has at least two copies in each mitogenome. A sequence alignment showed that the *trn* gene sequences display high similarities among these fungal species, such as for *trnN*, where 43 out of the 71/72 bp (61%) are in consensus in the 20 mitogenomes, and completely identical (100%) in *B. bassiana*, *B. pseudobassiana* and *C. brongniartii* (Figure 4B). A comparative analysis showed that the distribution of *trn* genes is variable in Pezizomycotina, but identical in the Order Hypocreales (Figure 4A). The *trn* clusters are conspicuously observed in these mitogenomes, as reports in previous studies [24-26]. Excepting the largest *trn* cluster (TEMMLAFKLQHM) conserved in all of the Pezizomycotina mitogenomes, the others are generally conserved at the Order level, such as (YDSN) and (VISWP) in Hypocreales, (KGDSW) in Sordariales, (KGDSWISP) in Eurotiales and (KGDSIWSP) in Onygenales, although minor differences exist owing to the lack of a *trn* or a transposition event (Figure 4A). The clustering of *trn* is suggested to be a unique characteristic of all Pezizomycotina [24].

Based on a display of the orthologous protein-coding genes, *trn* genes and rRNAs in the 20 mitogenomes (Figure 4A), a similar gene order can be observed in the Hypocreales mitogenomes, with the exception of *trnG*, which changed position in *Fusarium* species. The gene order is essentially identical in the seven invertebrate-parasitic fungi (*B. bassiana*, *B. pseudobassiana*, *C. brongniartii*, *L. muscarium*, *C. militaris*, *M. anisopliae* and *P. chlamydosporia*) and *H. jecorina*, as well as the four *Fusarium* fungi, as found in previous studies [24-26]. The same is also observed in Eurotiales and Onygenales. However, marked differences in gene order are observed between Sordariomycetes and Eurotiomycetes (Figure 4A). This indicates that in Pezizomycotina, which has closer phylogenetic relationships among its fungi, a greater number of identical gene orders are displayed, supporting the conclusion that the greater the identical gene orders the closer relationship they share [25].

Using CREx analysis [40], possible gene rearrangements in the 20 mitogenomes were predicted. A total of 21 transposition events were identified, 15 of which involve changes in *trn* position (Additional file 1: Figure S4). Among these, there are some rearrangement events that only occur in certain fungi (such as *trnL* in *A. tubingensis* from ‘LQ’ to ‘QL’ ), but no transposition event was found in Clavicipitaceae. Several events occur at the Order level, for example, the *trnG* location in Hypocreales involves at least three events (‘G nad6 V I S W P rnl T E M M L’ vs ‘nad6 V I S W P rnl T E M M L G’; ‘G nad6 V I S W P rnl T E M M’ vs ‘nad6 V I S W P rnl T E M M G’; ‘L G’ vs ‘G L’). Most of the rearrangements occur in Pezizomycotina, such as *trnH* (‘H M’ vs ‘M H’), *trnI* and *trnS* (‘I S W’ vs ‘W I S’, ‘S W’ vs ‘W S’), between Sordariomycetes and Eurotiomycetes (Figure 4A). Notably, many transposition events (13/21) occur in the region near *nad6* (Additional file 1: Figure S4), perhaps due to historical changes in gene order in this region. Although four types of rearrangement operations (i.e. transpositions, reverse transpositions, reversals and tandem-duplication-random-loss) were considered in the program CREx [40], only transposition events were predicted in our study, supporting the suggestion that the evolution of gene orders within Pezizomycotina is mostly driven by transpositions [12]. Even though the mechanisms of rearrangements are not fully understood, these rearrangement events may provide clues about the phylogeny and evolution of the fungal species in Pezizomycotina.

## Discussion

The mt genome represents a major model system in studies on evolutionary genomics, such as in animals [29] and fungi [12]. Here, the organization of the complete mt genome of *P. chlamydosporia* that is a promising BCA parasitizing nematode eggs is provided and its phylogenetic relationships to other invertebrate-pathogenic fungi are investigated. Synteny and phylogenetic analyses show that *P. chlamydosporia* is more closely related to *M. anisopliae* than to *L. muscarium* and other entomopathogenic fungi (Figure 2), even though both *P. chlamydosporia* and *L. muscarium* were once assigned to the same genus, *Verticillium*. Our results are consistent with previous results on the nuclear genome, in which the genome of *P. chlamydosporia* was found to be most closely related to entomopathogenic fungi *Metarhizium* spp. [11], as well as on the phylogenetic analyses of the *P. chlamydosporia* serine proteases [41,42]. Our phylogenetic results also show that fungi attributed to the same genus group together at first, such as the three species of *Beauveria* (*B. bassiana*, *B. pseudobassiana* and *C. brongniartii*, the latter is the teleomorph of *B. brongniartii* [43]), and the two species of *Lecanicillium* (*L. muscarium* and *C. militaris*, the teleomorph of *L. militaris* [44]). These five species then cluster into a clade because all of their teleomorphic forms are attributed to the same genus, *Cordyceps* [44,45]. Similarly, the four *Fusarium* species and the three *Aspergillus* species form two separate clades. According to our results, *P. chlamydosporia* and *M. anisopliae* also group together with a bootstrap value of 100%. The teleomorph of *P. chlamydosporia* is *Metacordyceps chlamydosporia*, but the teleomorph of *M. anisopliae* is unknown, therefore, we hypothesize that the teleomorphic form of *M. anisopliae* may also belong to the genus *Metacordyceps*.

Our phylogenetic tree showed that, in Hypocreales, all of the invertebrate-pathogenic fungi cluster together to form a monophyletic group, which is noticeably distinguished from a cluster comprising plant pathogens. This suggests that these invertebrate-pathogenic fungi have a most recent common ancestor (MRCA). Notably, in our phylogenetic tree, the fungus *H. jecorina* (the anamorphic name *Trichoderma reesei* [46]) groups together with the four *Fusarium* species, and is obviously outside the group entomopathogens. This is in agreement with some previous studies [47,48], but distinctly different from that in others [11,12,24,30,32], in which *H. jecorina* was within entomophagous group and more closely related to *M. anisopliae*. Because the results were obtained from both the total genomic data [11,48] and the mitogenomic data [12,24,30,32,47], we believe this may reflect to the different evolutionary rates of the nuclear and mt genomes or the much bigger data (number of genes) provided by the nuclear genome in comparison with mt genes. Considering *H. jecorina* that is attributed to the Family Hypocreaceae in fungal taxonomy, we think it is more reasonable that *H. jecorina* is outside the group entomopathogens, which belong to the Family Clavicipitaceae. Based on the phylogenetic relationship in our study, the evolution of fungi in Hypocreales may be speculated to have evolved from being parasitic on plants towards being parasitic on invertebrates. We think that a host habitat hypothesis can be used to explain the host shift in Hypocreales fungi, which suggests that host shifts tend to follow the host’s microhabitat or feeding habitat, resulting in a group of related endoparasites that exploit distantly related organisms at higher taxonomic levels [9]. Nikoh and Fukatsu reported that the entomoparasitic fungi of *Cordyceps* (Hypocreales) have an interkingdom host jumping from Animalia to Fungi by overlapping the ecological niches of the unrelated hosts [9]. Because most Hypocreales fungi grow naturally in soil, interkingdom host jumping events in Hypocreales might occur in an underground environment. It is thus worthwhile to study the evolution of Hypocreales fungi in depth in the future.

Among the ascomycete fungi, it was reported that the group I intron encoded version of *rps3* appears to have a rather complex evolutionary history [21]. However, the evolution of *rps3* in Hypocreales fungi has not been reported so far. In this study, our phylogenetic analysis shows that mitogenome *rps3* genes display an evolutionary pattern distinctly different from those inferred from the 14 mt protein-coding genes in Hypocreales fungi (Figure 2A). A JTT model showed a dN/dS value less than 1, indicating that the encoded protein is under functional constraints, i.e., natural selection is operating to minimize the number of amino acid changes, thereby maintaining the activity of the protein; a result similar to that found in a study with Ophiostomatoid fungi [21]. However, a site model analysis showed a clear signal of positive selection in these *rps3* sequences. Moreover, the dN/dS values obtained for all of the *rps3* sequences in Hypocreales - in relation to the reference sequence of *T. rubrum* - are also greater than 1, suggesting that positive selection acts on *rps3* in Hypocreales fungi. Such a positive selection on *rps3* was also observed previously in gymnosperms [49]. Although the special function of *rps3* in ascomycetes fungi is unclear, it is known that *rps3* plays a critical role in ribosome biogenesis and DNA repair in other eukaryotes [50]. Therefore, further investigation is needed to fully understand the evolution of fungal *rps3* genes.

As shown recently by the comparison of 38 complete fungal mt genomes from all the major phyla, remarkable variation is observed in genome size, gene order, composition of intergenic regions, repeats and introns [22]. In our study, we compared the sizes of the mt genomes from fungal genera belonging to Sordariomycetes and showed that, CRs, intronic and intergenic regions all contributed to genome size variation. However, the major contributor was the length of the intergenic regions or the intronic regions in the majority of the fungal mitogenomes (Additional file 1: Table S3). Our result indicates that, in Sordariomycetes, the length of intergenic regions is also an important contributor to fungal mitogenome size variation, the same as the length of introns.

A remarkable characteristic of fungal mitogenomes is that, similar to plant mitogenomes, fungal mitogenomes show signals of recombination. A comparison of 38 complete fungal mitogenomes (including the major fungal group) showed that the patterns of rearrangements may be explained by the combined influences of recombination, accumulated repeats, especially at intergenic regions, and to a lesser extent, mobile element dynamics [22]. In this study, we compared the *trn* in the 20 mitogenomes of Pezizomycotina fungi, and found that the gene order for all the protein- and rRNA-coding genes were principally conserved in Sordariomycetes, but *trn* clusters were conserved at the Order level (Figure 4A). Obvious differences of gene order and *trn* clusters were observed between Sordariomycetes and Eurotiomycetes. Our results support the previous reports that the Sordariomycetes and the Eurotiomycetes have highly conserved gene arrangements [22]. Moreover, several gene order rearrangement events in the 20 Pezizomycotina fungi mitogenomes were estimated by CREx analysis, and 21 transposition events in the mitogenomes were identified (Additional file 1: Figure S4). Out of these, 15 events displayed changes in *trn* order. Our results support the view that the evolution of gene order in Pezizomycotina is mostly driven by transpositions [12], although four operations of inversion, transposition, reverse-transposition and tandem-duplication-random-loss were considered in CREx program [27]. The observed transpositions also support the previous suggestion that *trn* genes should be considered as mobile elements involved in gene rearrangement [29,30]. Our results may provide an illustration of *trn* genes location changes in gene rearrangement in Pezizomycotina fungi mitogenomes.

## Conclusions

In this study, the complete and annotated mt genome sequence of *P. chlamydosporia* is provided. The relationships among the invertebrate pathogenic fungi in Hypocreales are determined. According to different model predictions, our results indicate that the *rps3* gene has experienced positive selection leading to a unique evolutionary pattern in Hypocreales. A comparison of the mitogenome sizes in Sordariomycetes shows that intergenic regions are as important as introns contributing to mitogenome size variation in Sordariomycete. Gene rearrangement analysis shows that the operation of transposition drives the rearrangement events of gene order in Pezizomycotina, and most of them display changes in *trn* order.

## Methods

### Fungal isolate

The fungal isolate of *P. chlamydosporia* stain 170 used in this study was originally isolated from RKN *Meloidogyne incognita* eggs and confirmed using ITS sequences. This isolate was deposited into the China General Microbiological Culture Collection Center (CGMCC, number 8860). The fungus was grown on potato dextrose agar at 28°C.

### DNA preparation, sequencing and assembly

Total DNA of *P. chlamydosporia* strain 170 was isolated from freeze-dried mycelium from liquid cultures following Fountaine’s description [51]. Three libraries with average insert sizes of 165 bp, 760 bp and 4,261 bp were constructed and sequenced using an Illumina Hiseq 2000 at BGI-Shenzhen (China). A total of 57,274,568 paired-end reads of the three libraries were produced (Additional file 1: Table S8). For these reads, ALLPATHS-LG revision 42305 [52] was used for assembly. From the assembled sequences, one 25,710 bp contig encoding several mt genes was discovered after an alignment to the NCBI NT database using BLASTN. The contig was then selected to amplify both ends of the DNA sequence with the primers (5′-GTACCTATTAACGGTACGGCTA-3′ and 5′-TTAGCCGAGGCAGAATCTGAGT-3′) using PCR technology and sequencing. A single 661 bp PCR product was generated. Using the obtained DNA sequence, a complete 25,615 bp mt genome of *P. chlamydosporia* was confirmed, and the sequence was submitted to NCBI (KF479445). The sequencing coverage of the contig was estimated by aligning the reads from a short insert library (165 bp) to the genome using BWA [53], and an average coverage of 1,058 × per base was identified. SNPs were investigated using mpileup and bcftools in the samtools package [54].

### Gene prediction and genome annotation

The mt genes were predicted by the method described in [15]. All the ORFs were obtained by searching for the mitogenome sequence in the Swiss-Prot [55] and NCBI NR databases using exonerate [56]. From the displayed high sequence similarities identified in the Swiss-Prot/ NR databases, 15 conserved mt genes (*cox1-3*, *cob*, *nad1-6*, *nad4L*, *atp6*, *atp8-9*, *rps3*) were discovered and annotated. The prediction software tRNAscan-SE v1.3.1 [57] was used to discover the *trn*. Additionally, the rfam_scan.pl program available in the Rfam database was used for *trn* and rRNA identification by searching for the mitogenome sequences in Rfam v11.0 [58]. The annotated *trn* genes from published mt genomes, such as those from *Hypocrea jecorina* mitogenome, were used as reference genes for prediction via BLASTN. A total of 23 *trn* genes were confirmed. Moreover, RepeatMasker with default parameters [59], EMBOSS einverted and palindrome [60] were used to identify the repeats in the mitogenomes, and repeat content was discovered. We also used 12 published *P. chlamydosporia* mt gene sequences to investigate the variance in the *P. chlamydosporia* mitogenome, including six coding segments in strain IMI 113169 (*nad3-atp9*, *atp6*, *rns*, *nad1*, *nad3*, *cox3*) [61,62], four genes in strain IMI 156157 (*rns*, *nad1*, *nad3*, *cox3*) [62], one *atp6* gene in strain CBS 101244 and one *atp6* gene in strain CBS 504.66 [10].

### Comparative analysis

A total of 21 fungal mitogenomes were used for the comparative analysis (Table 1). The genome sequences and annotations of 19 mitogenomes were obtained from NCBI genbank files, with the exception of the *N. crassa* sequence, which was downloaded from the Broad Institute [63]. The *N. crassa trn* were predicted using a method similar to that used for the *P. chlamydosporia trn* identification. The *trn* analyses were performed based on comparing the *trn* arrangements in the mitogenomes of 20 Pezizomycotina fungi, including 12 Hypocreales fungi (*P. chlamydosporia*, *M. anisopliae* [24], *B. bassiana* [26], *B. pseudobassiana*, *C. brongniartii* [61], *L. muscarium* [30], *C. militaris* [38], *H. jecorina* [64], *F. solani* [65], *F. graminearum* [65], *F. verticillioides* [65] and *F. oxysporum* [25] ), one Phyllachorales fungus (*V. dahliae* [32] ), one Sordariales fungus (*N. crassa*), three Eurotiales fungi (*A. fumigatus* [66], *A. niger* [39] and *A. tubingensis* [67] ), and three Onygenales fungi (*T. rubrum* [68], *E. floccosum* [69] and *P. brasiliensis* [70] ). The proteins of seven fungi were selected for codon usage analysis, including two Clavicipitaceae species (*M. anisopliae*, *P. chlamydosporia*), and one from each of the families Hypocreaceae (*H. jecorina*), Nectriaceae (*F. oxysporum*), Plectosphaerellaceae (*V. dahliae*), Sordariaceae (*N. crassa*) and Trichocomaceae (*A. fumigatus*) and using *C. parapsilosis* of Saccharomycotina as the outgroup for phylogeny comparisons. Five genomes displaying sizes with different variances were used for the size comparison. The rearrangement events in the mitogenomes were identified with CREx [40]. The gene orders of the 20 mitogenomes were uploaded to the CREx web server [71] which covers inversions, transpositions, reverse transpositions, and tandem-duplication-random-losses. In this study, only transposition operations were identified.

### Phylogenetic analysis

The 21 fungal mitogenomes were used for inferring a phylogenetic tree. In total, 14 mt genes (*cox1-3*, c*ob*, *nad1-6*, *nad4L*, *atp6*, and a*tp8-9*) were identified in the 21 organisms, however, two *nad2* (NCU16001, 235 aa; NCU16006, 584 aa) were encoded in *N. crassa* mitochondria, and both *nad2* genes had stop codons. Both of these *nad2* proteins showed a high level of sequence similarity to *nad2* in other fungi using BLASTP with a threshold of 1e-50. NCU16001 is shorter than NCU16006. A block (1–195 aa) of the sequence NCU16001 aligned to the NCU16006 block (1–233 aa) with an E-value of 2e-66. However, the size of NCU16006 is similar to the *nad2* of other Pezizomycotina species (such as *H. jecorina*), and NCU16006 is adjacent to *nad3*, which is a conserved unit in Pezizomycotina. To infer the true phylogeny, two groups of proteins (group I used 14 proteins in 21 species, including NCU16006 but not NCU16001 in *N. crassa*, and group II used 14 proteins in 20 species except *N. crassa*) were investigated through phylogenetic analysis, and the results displayed similar topologies.

The phylogenetic trees were constructed from both the nucleotide and amino acid sequences of the mt genes (Additional file 1: Table S9) using a ML method. A multiple sequence alignment of each gene was analysed using MUSCLE version 3.8.31 [72]. Certain poor alignment positions may have been saturated by multiple substitutions, and these regions may be obstacles to obtaining a reliable molecular phylogeny. The amino acid sequences of these core genes were concatenated, and the regions of poor alignment, including gaps, were removed using Gblocks version 0.91 with default parameters [73]. The unambiguously aligned portions of the amino acid sequences were obtained, and the corresponding nucleotide sequences were generated by running an in-house perl script. The best models for phylogenetic analysis of the nucleotide sequences were evaluated using jModelTest version 2.1.4 [74], which calculated 20 models. The evaluations generated by the Akaike Information Criterion (AIC) [75] and the Bayesian Information Criterion (BIC) [76] suggested that the General Time Reversible (GTR) [77] model coupled with rate variation among sites (+G) was the best model. Therefore, the GTR + G model was used for phylogenetic analysis. The nucleotide sequences were used to generate an ML tree with bootstrapping (1000 replicates) using MEGA version 6.06 [78] and PhyML version 3.1 [79]. Additionally, ProtTest version 3.4 [80] indicated that cpREV [81] combined with rate variation among sites (+G) and empirical frequencies (+F) was the best-fit model out of 120 models for the amino acid sequences, according to both AIC [75] and BIC [76]. The aligned amino acid sequences were used to construct the ML trees with 1000 bootstrap replicates using MEGA [78] and PhyML [79]. The topologies generated by both the nucleotide sequences and amino acids were similar to each other.

The *rps3* genes were identified and annotated in 20 fungi, and used to infer phylogeny. jModelTest [74] and ProtTest [80] were performed to select the best-fit models for the sequences of *rps3*. For the nucleotide sequences, the GTR + G model was used. For the amino acid sequences, the Jones-Taylor-Thornton (JTT) [82] model combining rate variation among sites (+G) and empirical frequencies (+F) was chosen. The two models (GTR + G, JTT + G + F) were evaluated with AIC [75] and BIC [76]. The ML trees of the sequences were constructed using MEGA [78] and PhyML [79]. The dN/dS ratio of *rps3* was analysed using CODEML with a JTT [82] model in PAML v4.7a [34], and site models M1 (neutral), M2 (selection), M7 (beta) and M8 (beta & ω) were used to detect the positive selection pressure on the genes. Although the values of dN/dS are less than or equal to 0.158, the LRT statistic for comparing M7 (lnL (log likelihood value) = −10,186.296) and M8 (lnL = −10,173.369) is 25.854 (2Δ = 2* (10,186.296-10,173.369) = 25.854), with a P-value of 2.432e-06 using the Chi-square test (with d.f. = 2). Furthermore, the dN/dS values of each *rps3* sequence with the *rps3* sequence in *T. rubrum* were calculated using DnaSP 5.10.1 [37] and CODEML [34].

### Data depositions

The mt genome sequence of *P. chlamydosporia* has been submitted to NCBI’s GenBank under the accession number: KF479445. And the phylogenetic data in this study has been submitted to TreeBASE [83].

## Additional file

Additional file 1: Table S1. Comparison of the assembled mitogenome sequences with the published *P. chlamydosporia* mt genes. **Table S2.** Gene and intergenic regions sizes from the complete mitogenomes of five Pezizomycotina fungal species. **Table S3.** The three primary contributors to mitogenome size variation. **Table S4.** Distinct codon usage for fungal mitogenome genes. **Table S5.** Codon usage bias statistics. **Table S6.** Nucleotide sequence lengths (bp) of the *rps3* gene and group I introns. **Table S7.** The dN/dS values of the *rps3* sequences. **Table S8.** Summary of the sequenced data of *P. chlamydosporia*. **Table S9.** Proteins used for phylogenetic analyses are shown. **Figure S1.** Distribution of the genes and repeats in the mitogenomes of *P. chlamydosporia*, *M. anisopliae* and *L. muscarium*. **Figure S2.** Synteny between *P. chlamydosporia* and *B. bassiana*, *V. dahliae*, *A. fumigatus*. **Figure S3.** Evolutionary characteristics of *rps3*. **Figure S4.** Putative transposition events in the mitogenomes of 20 fungi detected by the use of CREx algorithm.

## Abbreviations

BCA: Biological control agent
ITS: Internal transcribed spacer
mt: Mitochondrial
RKN: Root-knot nematode
*cox*: Cytochrome c oxidase subunit
*cob*: Apocytochrome b
*atp*: ATP synthase subunit
*nad*: Subunit of NADH dehydrogenase
*rps3*: Ribosomal protein S3
*rns*: Small ribosomal RNA
*rnl*: Large ribosomal RNA
*trn*: Transfer RNA genes
SNP: Single nucleotide polymorphism
CDS: Coding DNA sequence
CR: Coding region
LRT: Likelihood ratio test
d.f.: Degrees of freedom
ML: Maximum likelihood
MRCA: Most recent common ancestor
