# Supplementary material for: Analysis of the complete mitochondrial genome of Pochonia chlamydosporia suggests a close relationship to the invertebrate-pathogenic fungi in Hypocreales
Source: BMC Microbiol. 2015 Jan 31;15:5. doi: 10.1186/s12866-015-0341-8 (PMC4360972; doi:10.1186/s12866-015-0341-8)
Supplement: Additional file 1: Table S1. — Comparison of the assembled mitogenome sequences with the published P. chlamydosporia mt genes. Table S2. Gene and intergenic regions sizes from the complete mitogenomes of five Pezizomycotina fungal species. Table S3. The three primary contributors to mitogenome size variation. Table S4. Distinct codon usage for fungal mitogenome genes. Table S5. Codon usage bias statistics. Table S6. Nucleotide sequence lengths (bp) of the rps3 gene and group I introns. Table S7. The dN/dS values of the rps3 sequences. Table S8. Summary of the sequenced data of P. chlamydosporia. Table S9. Proteins used for phylogenetic analyses are shown. Figure S1. Distribution of the genes and repeats in the mitogenomes of P. chlamydosporia, M. anisopliae and L. muscarium. Figure S2. Synteny between P. chlamydosporia and B. bassiana, V. dahliae, A. fumigatus. Figure S3. Evolutionary characteristics of rps3. Figure S4. Putative transposition events in the mitogenomes of 20 fungi detected by the use of CREx algorithm. [file 12866_2015_341_MOESM1_ESM.pdf]

# Analysis of the complete mitochondrial genome of *Pochonia chlamydosporia* suggests a close relationship to the invertebrate-pathogenic fungi in Hypocreales

**Runmao Lin[1†], Chichuan Liu[1†], Baoming Shen[1,2], Miao Bai[3], Jian Ling[1], Guohua Chen[1], Zhenchuan Mao[1], Xinyue Cheng[4*] and Bingyan Xie[1*]**

**Additional file 1**

**Supplementary Tables**

**Table S1.** Comparison of the assembled mitogenome sequences with the published *P. chlamydosporia* mt genes. **Table S2.** Gene and intergenic regions sizes from the complete mitogenomes of five Pezizomycotina fungal species. **Table S3.** The three primary contributors to mitogenome size variation. **Table S4.** Distinct codon usage for fungal mitogenome genes. **Table S5.** Codon usage bias statistics. **Table S6.** Nucleotide sequence lengths (bp) of the *rps3* gene and group I introns. **Table S7.** The dN/dS values of the *rps3* sequences. **Table S8.** Summary of the sequenced data of *P. chlamydosporia*. **Table S9.** Proteins used for phylogenetic analyses are shown.

**Supplementary Figures**

**Figure S1.** Distribution of the genes and repeats in the mitogenomes of *P. chlamydosporia*, *M. anisopliae* and *L. muscarium*. **Figure S2.** Synteny between *P. chlamydosporia* and *B. bassiana*, *V. dahliae*, *A. fumigatus*. **Figure S3.** Evolutionary characteristics of *rps3*. **Figure S4.** Putative transposition events in the mitogenomes of 20 fungi detected by the use of CREx algorithm.

**Supplementary Tables**

**Table S1. Comparison of the assembled mitogenome sequences with the published *P. chlamydosporia* mt genes.** BLASTN were used for alignment analyses.

| Accession | Length (bp) | Identity | Matched (%) | E_value | Description |
|---|---|---|---|---|---|
| FJ973100.1 | 323 | 0.97 | 100.00 | 1.00E-170 | *Pochonia chlamydosporia* strain IMI 113169 NADH dehydrogenase subunit 3 (*nad3*) and ATP synthase F0 subunit 9 (*atp9*) genes, partial cds |
| FJ973052.1 | 452 | 0.99 | 96.90 | 0 | *Pochonia chlamydosporia* strain IMI 113169 ATP synthase F0 subunit 6 (*atp6*) gene, partial cds; and small subunit ribosomal RNA (*rns*) gene, partial sequence |
| AY556048.1 | 439 | 0.84 | 97.27 | 9.00E-83 | *Pochonia chlamydosporia* strain IMI 113169 small subunit ribosomal RNA (*rns*) gene, partial sequence |
| AY556020.1 | 158 | 0.95 | 100.00 | 7.00E-72 | *Pochonia chlamydosporia* strain IMI 113169 NADH dehydrogenase subunit 3 (*nad3*) gene, partial cds |
| AY555993.1 | 479 | 0.98 | 100.00 | 0 | *Pochonia chlamydosporia* strain IMI 113169 NADH dehydrogenase subunit 1 (*nad1*) gene, partial cds |
| AY555938.1 | 363 | 0.99 | 100.00 | 0 | *Pochonia chlamydosporia* strain IMI 113169 cytochrome oxidase subunit III (*cox3*) gene, partial cds |
| AY556047.1 | 501 | 0.99 | 100.00 | 0 | *Pochonia chlamydosporia* strain IMI 156157 small subunit ribosomal RNA (*rns*) gene, partial sequence |
| AY556019.1 | 158 | 0.98 | 100.00 | 8.00E-84 | *Pochonia chlamydosporia* strain IMI 156157 NADH dehydrogenase subunit 3 (*nad3*) gene, partial cds |
| AY555992.1 | 479 | 0.97 | 100.00 | 0 | *Pochonia chlamydosporia* strain IMI 156157 NADH dehydrogenase subunit 1 (*nad1*) gene, partial cds |
| AY555937.1 | 363 | 0.99 | 100.00 | 0 | *Pochonia chlamydosporia* strain IMI 156157 cytochrome oxidase subunit III (*cox3*) gene, partial cds |
| EF469008.1 | 633 | 0.99 | 100.00 | 0 | *Cordyceps chlamydosporia* strain CBS 101244 ATP synthase subunit 6 (*atp6*) gene, partial cds |
| EF469040.1 | 614 | 0.98 | 100.00 | 0 | Pochonia chlamydosporia strain CBS 504.66 ATP synthase subunit 6 (*atp6*) gene, partial cds |

**Table S2. Gene and intergenic regions sizes from the complete mitogenomes of five Pezizomycotina fungal species.** Some genes share overlap sequences, and length of overlap sequences among coding regions (CRs) is calculated once.

| Species | Length (bp) | CRs (bp) | tRNAs (bp) | rRNAs (bp) | Introns (bp) | Intergenic (bp) | Genes have Intron |
|---|---|---|---|---|---|---|---|
| *M. anisopliae* | 24,673 | 14,562 (59.02%) | 1,786 (7.24%) | 6,240 (25.29%) | 313 (1.27%) | 3,468 (14.06%) | *rnl* |
| *P. chlamydosporia* | 25,615 | 14,385 (56.16%) | 1,697 (6.63%) | 6,232 (24.33%) | 335 (1.31%) | 4,618 (18.03%) | *rnl* |
| *F. oxysporum* | 34,477 | 15,399 (44.66%) | 1,865 (5.41%) | 6,470 (18.77%) | 1,397 (4.05%) | 11,074 (32.12%) | *rnl*, *nad5* |
| *H. jecorina* | 42,130 | 19,830 (47.07%) | 1,937 (4.60%) | 5,904 (14.01%) | 10,049 (23.85%) | 7,261 (17.23%) | *rnl*, *cox2*, *cob*, *cox1* |
| *N. crassa* | 64,840 | 27,711 (42.74%) | 2,074 (3.20%) | 7,179 (11.07%) | 6,465 (9.97%) | 23,155 (35.71%) | *rnl*, *nad3*, *nad4L*, NCU16011, NCU16015, *nad1*, *nad4*, *atp6*, *nad5*, *cob* |

Note: 1. Overlaps between genes:

*H. jecorina*: *nad4L*, *nad5*: 1 bp (i.e., 1 bp of overlap sequence between *nad4L* and *nad5*); *cox1*, unknown gene: 690 bp.

*N. crassa*: *nad2*, NCU16002: 1 bp; *nad4L*, NCU16009: 1359 bp; *nad4L*, NCU16010: 1 bp; *nad4L*, NCU16011: 1 bp; *nad4L*, *nad5*: 1 bp; NCU16010, NCU16011: 1602; NCU16010, *nad5*: 1602 bp; NCU16011, *nad5*: 3047; *cob*, NCU16014: 1 bp; *cob*, NCU16015: 1 bp; NCU16014, NCU16015: 1347; *cox1*, NCU16017: 38 bp; *nad1*, NCU16019: 1416 bp; *nad4*, NCU16021: 2007 bp; *atp6*, NCU16026: 1443 bp.

**Table S3. The three primary contributors to mitogenome size variation.** Comparisons were performed pairwise and percentages are generated. The percentage is generated by calculating length of variant regions/length of mitogenome size variation and the negative number is ignored. For example, the size of *N. crassa* is 40,167 bp larger than that of *M. anisoplia*e and the intergenic regions (IRs) of *N. crassa* is 19,678 bp larger than those of *M. anisoplia*e mt genome, therefore 49% = 19,678/40,167. MA, *M. anisoplia*e; PC, *P. chlamydosporia*; HJ, *H. jecorina*; FO, *F. oxysporum*; NC, *N. crassa*; CRs, coding regions; IRs, intergenic regions.

| | **MA** | | | **PC** | | | **FO** | | | **HJ** | | |
|---|---|---|---|---|---|---|---|---|---|---|---|---|
| | **1** | **2** | **3** | **1** | **2** | **3** | **1** | **2** | **3** | **1** | **2** | **3** |
| NC | IRs 49% | CRs 33% | Introns 15% | IRs 47% | CRs 34% | Introns 16% | CRs 41% | IRs 40% | Introns 17% | IRs 70% | CRs 35% | -- |
| HJ | Introns 56% | CRs 30% | IRs 22% | Introns 59% | CRs 33% | IRs 16% | Introns 113% | CRs 58% | -- | | | |
| FO | IRs 78% | Introns 11% | CRs 9% | IRs 73% | Introns 12% | CRs 11% | | | | | | |
| PC | IRs 122% | Introns 22% | -- | | | | | | | | | |

**Table S4. Distinct codon usage for fungal mitogenome genes.** MA, *M. anisopliae*; PC, *P. chlamydosporia*; HJ, *H. jecorina*; FO, *F. oxysporum*; VD, *V. dahliae*; NC, *N. crassa*; AF, *A. fumigatus*.

| AA | Codon | MA (%) | PC (%) | HJ (%) | FO (%) | VD (%) | NC (%) | AF (%) |
|---|---|---|---|---|---|---|---|---|
| F | TTC | 3.96 | 4.19 | 2.78 | 2.57 | 2.65 | 1.73 | 2.52 |
| I | ATA | 7.52 | 7.3 | 6.93 | 7.91 | 6.5 | 5.33 | 5.6 |
| L | CTC | 0.04 | 0 | 0.11 | 0.04 | 0.02 | 0.27 | 0.03 |
| | CTG | 0.04 | 0 | 0.18 | 0.18 | 0 | 0.33 | 0.03 |
| | TTA | 12.73 | 12.51 | 9.95 | 11.09 | 12.28 | 8.29 | 12.27 |
| R | AGG | 0.19 | 0 | 0.02 | 0.06 | 0.04 | 0.33 | 0.07 |
| | CGA | 0 | 0.02 | 0.02 | 0 | 0.04 | 0.23 | 0 |
| | CGC | 0.02 | 0 | 0.02 | 0 | 0 | 0.09 | 0 |
| | CGG | 0.06 | 0 | 0.05 | 0.02 | 0.02 | 0.07 | 0.02 |
| U | TAG | 0 | 0.02 | 0.08 | 0.06 | 0.02 | 0.1 | 0.05 |
| V | GTA | 4.18 | 4.28 | 3.27 | 3.37 | 3.45 | 2.5 | 3.52 |
| W | TGG | 0.12 | 0 | 0.08 | 0.04 | 0 | 0.18 | 0.1 |

**Table S5. Codon usage bias statistics.** MA, *M. anisopliae*; PC, *P. chlamydosporia*; HJ, *H. jecorina*; FO, *F. oxysporum*; VD, *V. dahliae*; NC, *N. crassa*; AF, *A. fumigatus*.

| Codon | MA (%) | PC (%) | HJ (%) | FO (%) | VD (%) | NC (%) | AF (%) |
|---|---|---|---|---|---|---|---|
| A/T | 83.05 | 84.27 | 85.47 | 85.28 | 88.78 | 81.42 | 90.17 |

**Table S6. Nucleotide sequence lengths (bp) of the *rps3* gene and group I introns.**

| Species | *rps3* | Group I intron |
|---|---|---|
| *C. brongniartii* | 1,416 | 1,926 |
| *B. bassiana* | 1,389 | 1,876 |
| *B. pseudobassiana* | 1,389 | 1,887 |
| *C. militaris* | 1,323 | 1,828 |
| *L. muscarium* | 1,332 | 1,709 |
| *P. chlamydosporia* | 1,317 | 1,652 |
| *M. anisopliae* | 1,383 | 1,696 |
| *H. jecorina* | 1,425 | 1,535 |
| *F. solani* | 1,536 | 2,061 |
| *F. graminearum* | 1,482 | 1,992 |
| *F. oxysporum* | 1,341 | 1,728 |
| *F. verticillioides* | 1,545 | 2,445 |
| *V. dahlia* | 1,425 | 1,774 |
| *N. crassa* | 1,527 | 2,266 |
| *A. fumigatus* | 1,233 | 1,601 |
| *A. tubingensis* | 1,218 | 1,794 |
| *A. niger* | 1,218 | 1,800 |
| *P. brasiliensis* | 1,212 | 1,685 |
| *T. rubrum* | 1,140 | 1,539 |
| *E. floccosum* | 1,140 | 1,547 |

**Table S7. The dN/dS values of the *rps3* sequences.** The dN/dS values are generated based on comparative analysis with the *rps3* of *T. rubrum*. FO, *F. oxysporum*; MA, *M. anisopliae*; PC, *P. chlamydosporia*; HJ, *H. jecorina*; FS, *F. solani*; FG, *Fusarium graminearum*; FV, *F. verticillioides*; CM, *C. militaris*; LM, *L. muscarium*; CB, *C. brongniartii*; BB, *B. bassiana*; BP, *B. pseudobassiana*; VD, *V. dahliae*; NC, *N. crassa*; AF, *A. fumigatus*; AT, *A. tubingensis*; AN, *A. niger*; EF, *E. floccosum*; TR, *T. rubrum*; PB, *P. brasiliensis*.

| | | **CODEML (version 4.7a)** | | | **DNASP (version 5.10.1)** | | |
|---|---|---|---|---|---|---|---|
| **Seq1** | **Seq2** | **dN** | **dS** | **dN/dS** | **dN** | **dS** | **dN/dS** |
| TR | FO | 0.7752 | 0.5873 | 1.3199 | 0.7804 | 0.5742 | 1.3591 |
| TR | MA | 0.8847 | 0.5919 | 1.4947 | 0.8917 | 0.5772 | 1.5449 |
| TR | PC | 0.7551 | 0.5438 | 1.3885 | 0.7599 | 0.5324 | 1.4273 |
| TR | HJ | 0.7726 | 0.5837 | 1.3236 | 0.7779 | 0.5706 | 1.3633 |
| TR | FV | 0.7499 | 0.5520 | 1.3584 | 0.7556 | 0.5383 | 1.4037 |
| TR | FG | 0.7327 | 0.5748 | 1.2748 | 0.7380 | 0.5611 | 1.3153 |
| TR | FS | 0.7460 | 0.5227 | 1.4272 | 0.7517 | 0.5100 | 1.4739 |
| TR | BB | 0.7873 | 0.4746 | 1.6589 | 0.7926 | 0.4650 | 1.7045 |
| TR | BP | 0.8002 | 0.4974 | 1.6089 | 0.8061 | 0.4863 | 1.6576 |
| TR | CB | 0.8178 | 0.4914 | 1.6642 | 0.8252 | 0.4783 | 1.7253 |
| TR | CM | 0.8278 | 0.5704 | 1.4512 | 0.8337 | 0.5578 | 1.4946 |
| TR | LM | 0.7917 | 0.5268 | 1.5030 | 0.7977 | 0.5142 | 1.5513 |
| TR | VD | 0.7866 | 0.4585 | 1.7158 | 0.7922 | 0.4488 | 1.7652 |
| TR | NC | 0.7281 | 0.5904 | 1.2333 | 0.7308 | 0.5830 | 1.2535 |
| TR | AN | 0.3703 | 0.3908 | 0.9474 | 0.3718 | 0.3846 | 0.9667 |
| TR | AT | 0.3776 | 0.3860 | 0.9783 | 0.3792 | 0.3798 | 0.9984 |
| TR | AF | 0.3701 | 0.4511 | 0.8205 | 0.3724 | 0.4397 | 0.8469 |
| TR | EF | 0.0401 | 0.1309 | 0.3061 | 0.0402 | 0.1297 | 0.3099 |
| TR | PB | 0.4697 | 0.5382 | 0.8728 | 0.4730 | 0.5229 | 0.9046 |

**Table S8. Summary of the sequenced data of *P. chlamydosporia*.** Three libraries were constructed and sequenced using Illumina HiSeq 2000 technology.

| **Insert Size (bp)** | **# of reads** | **Reads Length (bp)** | **# of Bases** |
|---|---|---|---|
| 165 | 42,331,564 | (90, 90) | 3,809,840,760 |
| 760 | 47,699,680 | (90, 90) | 4,292,971,200 |
| 4,261 | 24,517,892 | (49, 49) | 1,201,376,708 |
| Total | | | 9,304,188,668 |

**Table S9. Proteins used for phylogenetic analyses are shown.** The accession numbers of these proteins are obtained from the GenBank files introduced in Table 1. BB, *B. bassiana*; BP, *B. pseudobassiana*; CB, *C. brongniartii*; LM, *L. muscarium*; CM, *C. militaris*; MA, *M. anisopliae*; PC, *P. chlamydosporia*; HJ, *H. jecorina*; FS, *F. solani*; FG, *F. graminearum*; FO, *F. oxysporum*; FV, *F. verticillioides*; VD, *V. dahliae*; NC, *N. crassa*; AN, *A. niger*; AT, *A. tubingensis*; AF, *A. fumigatus*; PB, *P. brasiliensis*; TR, *T. rubrum*; EF, *E. floccosum*; CP, *C. parapsilosis*.

| Species | *rps3* | *nad1* | *nad2* | *nad3* | *nad4* | *nad4L* | *nad5* | *nad6* | *atp6* | *atp8* | *atp9* | *cox1* | *cox2* | *cox3* | *cob* |
|---|---|---|---|---|---|---|---|---|---|---|---|---|---|---|---|
| BB | ABY61762.1 | ABY61755.1 | ABY61748.1 | ABY61749.1 | ABY61756.1 | ABY61752.1 | ABY61761.1 | ABY61760.1 | ABY61758.1 | ABY61757.1 | ABY61750.1 | ABY61754.1 | ABY61751.1 | ABY61759.1 | ABY61753.1 |
| BP | YP_008758054.1 | YP_008758063.1 | YP_008758055.1 | YP_008758056.1 | YP_008758064.1 | YP_008758059.1 | YP_008758060.1 | YP_008758068.1 | YP_008758066.1 | YP_008758065.1 | YP_008758057.1 | YP_008758062.1 | YP_008758058.1 | YP_008758067.1 | YP_008758061.1 |
| CB | YP_002213593.1 | YP_002213605.1 | YP_002213594.1 | YP_002213595.1 | YP_002213607.1 | YP_002213598.1 | YP_002213599.1 | YP_002213611.1 | YP_002213609.1 | YP_002213608.1 | YP_002213596.1 | YP_002213602.1 | YP_002213597.1 | YP_002213610.1 | YP_002213600.1 |
| LM | AAO14657.1 | AAL27687.1 | AAO14658.1 | AAO14659.1 | AAO14666.1 | AAO14662.1 | AAO14663.1 | AAO14669.1 | AAO14668.1 | AAO14667.1 | AAO14660.1 | AAO14665.1 | AAO14661.1 | AAL27688.1 | AAO14664.1 |
| CM | YP_008815517.1 | YP_008815526.1 | YP_008815518.1 | YP_008815519.1 | YP_008815527.1 | YP_008815522.1 | YP_008815523.1 | YP_008815531.1 | YP_008815529.1 | YP_008815528.1 | YP_008815520.1 | YP_008815525.1 | YP_008815521.1 | YP_008815530.1 | YP_008815524.1 |
| MA | AAW58815.1 | AAW58824.1 | AAW58816.1 | AAW58817.1 | AAW58825.1 | AAW58820.1 | AAW58821.1 | AAW58829.1 | AAW58827.1 | AAW58826.1 | AAW58818.1 | AAW58823.1 | AAW58819.1 | AAW58828.1 | AAW58822.1 |
| PC | AGY95315.1 | AGY95324.1 | AGY95316.1 | AGY95317.1 | AGY95310.1 | AGY95320.1 | AGY95321.1 | AGY95314.1 | AGY95312.1 | AGY95311.1 | AGY95318.1 | AGY95323.1 | AGY95319.1 | AGY95313.1 | AGY95322.1 |
| HJ | AAL74166.1 | AAL74169.1 | AAL74165.1 | AAL74174.1 | AAL74179.1 | AAL74175.1 | AAL74164.1 | AAL74172.1 | AAL74171.1 | AAL74177.1 | AAL74176.1 | AAL74182.1 | AAL74180.1 | AAL74170.1 | AAL74181.1 |
| FS | YP_005088098.1 | YP_005088119.1 | YP_005088100.1 | YP_005088102.1 | YP_005088120.1 | YP_005088105.1 | YP_005088106.1 | YP_005088127.1 | YP_005088123.1 | YP_005088122.1 | YP_005088103.1 | YP_005088109.1 | YP_005088104.1 | YP_005088125.1 | YP_005088108.1 |
| FG | YP_001249297.1 | YP_001249337.1 | YP_001249299.1 | YP_001249304.1 | YP_001249339.1 | YP_001249312.1 | YP_001249314.1 | YP_001249346.1 | YP_001249341.1 | YP_001249340.1 | YP_001249306.1 | YP_001249323.1 | YP_001249308.1 | YP_001249343.1 | YP_001249316.1 |
| FO | AAX21823.1 | AAX21833.1 | AAX21824.1 | AAX21825.1 | AAX21834.1 | AAX21828.1 | AAX21829.1 | AAX21838.1 | AAX21836.1 | AAX21835.1 | AAX21826.1 | AAX21832.1 | AAX21827.1 | AAX21837.1 | AAX21831.1 |
| FV | YP_005088212.1 | YP_005088226.1 | YP_005088214.1 | YP_005088216.1 | YP_005088228.1 | YP_005088219.1 | YP_005088220.1 | YP_005088232.1 | YP_005088230.1 | YP_005088229.1 | YP_005088217.1 | YP_005088222.1 | YP_005088218.1 | YP_005088231.1 | YP_005088221.1 |
| VD | ABC60419.1 | ABC60422.1 | ABC60420.1 | ABC60421.1 | ABC60423.1 | ABC60430.1 | ABC60431.1 | ABC60427.1 | ABC60425.1 | ABC60424.1 | ABC60428.1 | ABC60433.1 | ABC60429.1 | ABC60426.1 | ABC60432.1 |
| NC* | NCU16005 | NCU16018 | NCU16006 | NCU16007 | NCU16020 | NCU16008 | NCU16012 | NCU16004 | NCU16025 | NCU16024 | NCU16027 | NCU16016 | NCU16028 | NCU16003 | NCU16013 |
| AN | rps3_AN** | YP_337877.1 | YP_337891.1 | YP_337887.1 | YP_337880.1 | YP_337889.1 | YP_337890.1 | YP_337883.1 | YP_337882.1 | YP_337881.1 | YP_337886.1 | YP_337885.1 | YP_337888.1 | YP_337884.1 | YP_337876.1 |
| AT | rps3_AT*** | YP_398768.1 | YP_398782.1 | YP_398778.1 | YP_398771.1 | YP_398780.1 | YP_398781.1 | YP_398774.1 | YP_398773.1 | YP_398772.1 | YP_398777.1 | YP_398776.1 | YP_398779.1 | YP_398775.1 | YP_398767.1 |
| AF | AFE02877.1 | AFE02871.1 | AFE02889.1 | AFE02885.1 | AFE02872.1 | AFE02887.1 | AFE02888.1 | AFE02875.1 | AFE02874.1 | AFE02873.1 | AFE02884.1 | AFE02880.1 | AFE02886.1 | AFE02876.1 | AFE02870.1 |
| PB | YP_537113.1 | YP_537107.1 | YP_537103.1 | YP_537106.1 | YP_537108.1 | YP_537118.1 | YP_537102.1 | YP_537111.1 | YP_537110.1 | YP_537109.1 | YP_537116.1 | YP_537114.1 | YP_537117.1 | YP_537112.1 | YP_537104.1 |
| TR | YP_002970776.1 | YP_002970787.1 | YP_002970784.1 | YP_002970786.1 | YP_002970788.1 | YP_002970782.1 | YP_002970783.1 | YP_002970790.1 | YP_002970789.1 | YP_002970775.1 | YP_002970780.1 | YP_002970779.1 | YP_002970781.1 | YP_002970774.1 | YP_002970785.1 |
| EF | YP_313620.1 | YP_313638.1 | YP_313633.1 | YP_313637.1 | YP_313639.1 | YP_313629.1 | YP_313630.1 | YP_313641.1 | YP_313640.1 | YP_313619.1 | YP_313627.1 | YP_313625.1 | YP_313628.1 | YP_313618.1 | YP_313634.1 |
| CP | -- | NP_943644.1 | NP_943631.1 | NP_943630.1 | NP_943641.2 | NP_943646.1 | NP_943647.1 | NP_943643.1 | NP_943649.1 | NP_943648.1 | NP_943642.1 | NP_943636.1 | NP_943635.1 | NP_943645.1 | NP_943632.1 |

Note: *, the protein IDs are obtained from the Broad Institute; **, ***, these two *rps3* genes (rps3_AN and rps3_AT) are predicted based on sequences of homologous *rps3* gene (AFE02877.1) in AF. The location of rps3_AN and rps3_AT in the AN mitogenome (NC_007445) and AT mitogenome (NC_007597) are from 16,403 to 17,620 bp and from 16,536 to 17,753 bp, respectively.

## Supplementary Figures

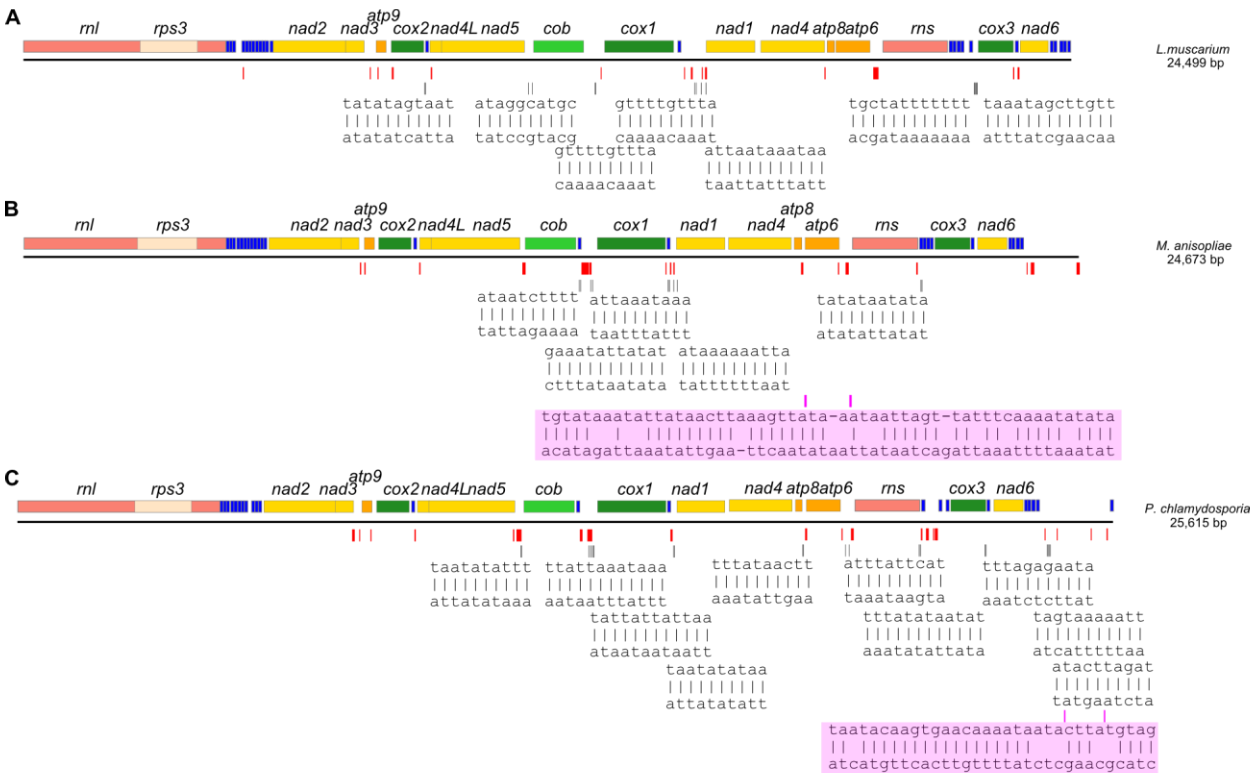


**Figure S1. Distribution of the genes and repeats in the mitogenomes of *P. chlamydosporia*, *M. anisopliae* and *L. muscarium*.** *trn* genes, tandem repeats, inverted repeats and palindromes are marked in blue, red, pink and grey, respectively. The inverted repeat sequences (such as the pair sequences of "tatatagtaat" from 9,382 bp to 9,392 and "atatatcatta" from 9,403 bp to 9,393 bp) and palindromic sequences are shown. In *L. muscarium*, tandem repeats and palindromes account for 532 bp and 156 bp, respectively; and there are 24 overlaps between tandem repeats and palindromes. In *M. anisopliae*, tandem repeats, inverted repeats and palindromes account for 854 bp, 109 bp and 108 bp, respectively; and 86 bp overlaps between tandem repeats and palindromes are found. In *P. chlamydosporia*, tandem repeats, inverted repeats and palindromes account for 948 bp, 64 bp and 172 bp, respectively; and there are 123 bp overlap sequences.

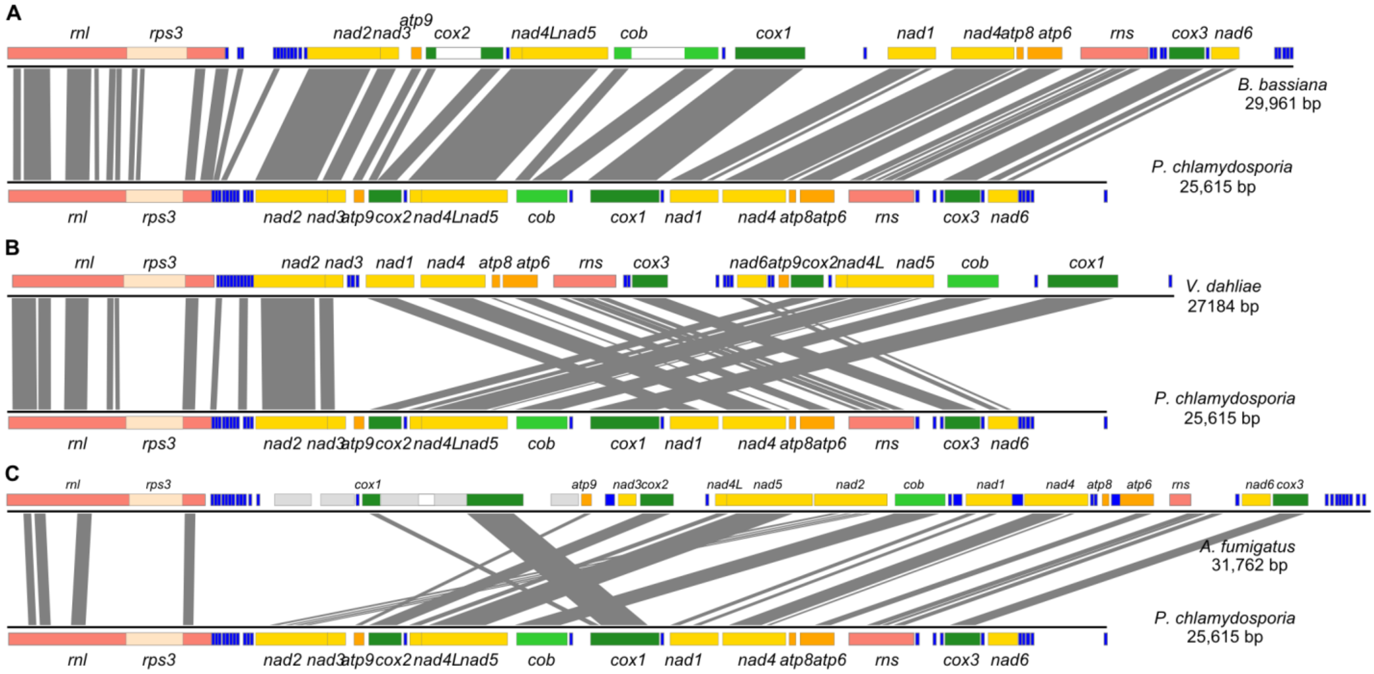


**Figure S2. Synteny between *P. chlamydosporia* and *B. bassiana*, *V. dahliae*, *A. fumigatus*.** The syntenic regions are identified by BLASTN analysis using E-value cutoff of 1e-5. Alignment blocks in size < 100 bp are not shown. The blue and gray blocks represent *trn* genes and predicted ORFs, respectively.

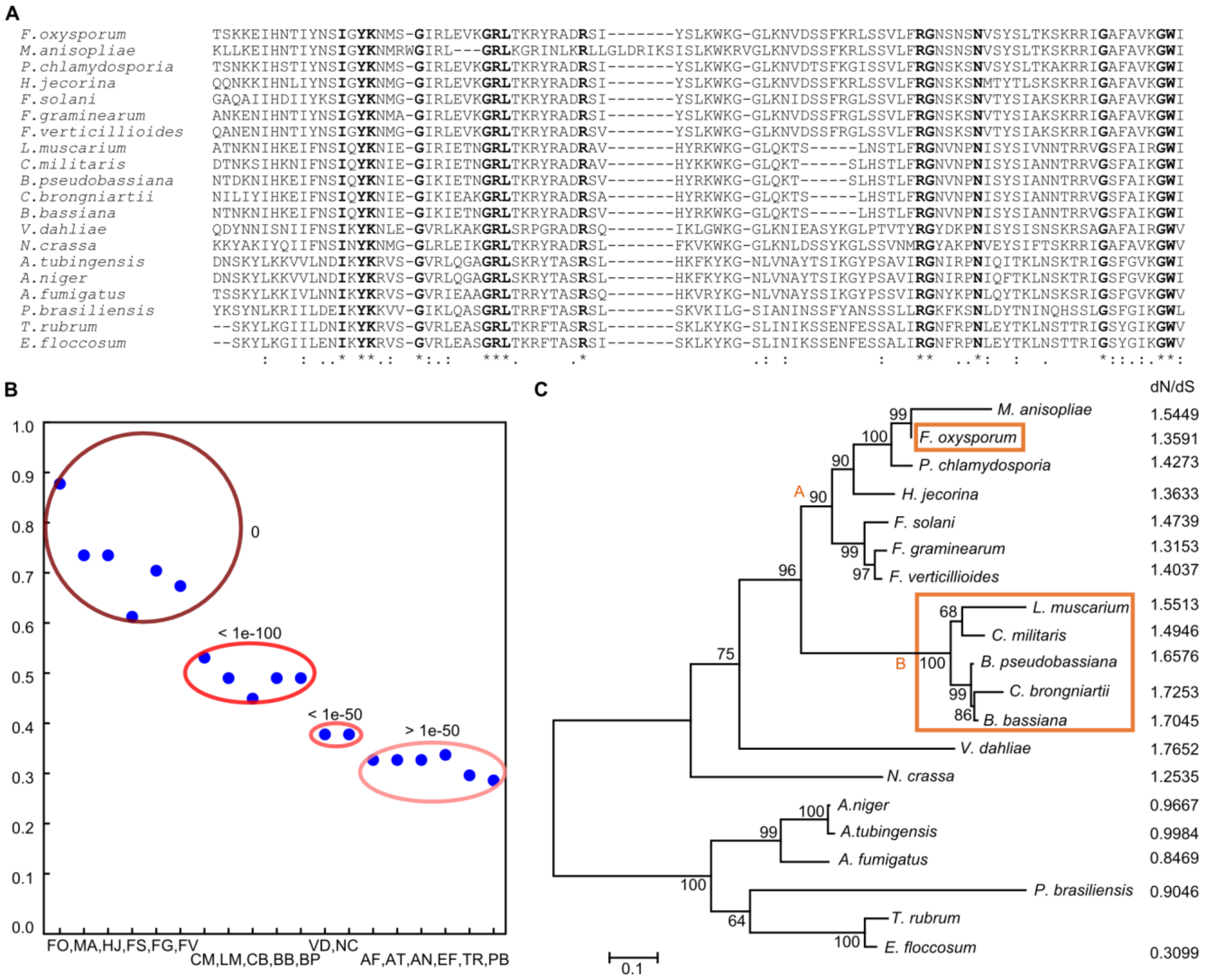


**Figure S3. Evolutionary characteristics of *rps3*. A). Alignment of C-terminal domain of ribosomal protein S3.** "*" indicates identical aa residues, which are marked in bold; ":" indicates highly conserved aa residues (scoring > 0.5); and "." shows weakly similar properties (scoring =< 0.5). **B). High *rps3* sequence similarity in four distinct groups of fungi.** Four groups of E-values are displayed in four circles. FO, *F. oxysporum*; MA, *M. anisopliae*; HJ, *H. jecorina*; FS, *F. solani*; FG, *Fusarium graminearum*; FV, *F. verticillioides*; CM, *C. militaris*; LM, *L. muscarium*; CB, *C. brongniartii*; BB, *B. bassiana*; BP, *B. pseudobassiana*; VD, *V. dahliae*; NC, *N. crassa*; AF, *A. fumigatus*; AT, *A. tubingensis*; AN, *A. niger*; EF, *E. floccosum*; TR, *T. rubrum*; PB, *P. brasiliensis*. **C). The Maximum Likehood (ML) tree of *rps3* from 20 fungal mt genomes.** The branches representing *F. oxysporum* and clade B are different from the genomic tree. The dN/dS values are calculated using DNASP (version version 5.10.1) based on comparative analysis with the *rps3* of *T. rubrum*.

| | |
|---|---|
| t1 (2) | rns Y D S N cox3 G nad6 V I S W P rnl T E M M L A F K L Q H M nad2 nad3 atp9 cox2 R nad4L nad5 R cob C cox1 R nad1 nad4 atp8 atp6 |
| | 1 15 |
| | rns Y D S N cox3 nad6 V I S W P rnl T E M M L G A F M K L Q H nad2 nad3 atp9 cox2 R nad4L nad5 R cob C cox1 R nad1 nad4 atp8 atp6 |
| t2 (1) | rns Y D S N cox3 G nad6 V I S W P rnl T E M M L A F K L Q H M nad2 nad3 atp9 cox2 R nad4L nad5 R cob C cox1 R nad1 nad4 atp8 atp6 |
| | 2 |
| | rns Y D S N cox3 nad6 V I S W P rnl T E M M G L G A F K L Q H M nad2 nad3 atp9 cox2 R R Y nad4L nad5 R cob C cox1 R nad1 nad4 atp8 atp6 |
| t3 (1) | rns Y D S N cox3 G nad6 V I S W P rnl T E M M L A F K L Q H M nad2 nad3 atp9 cox2 R nad4L nad5 R cob C cox1 R nad1 nad4 atp8 atp6 |
| | 4 |
| | rns Y N cox3 K G D S W nad6 V I S P rnl T E M M L A F M L Q H M nad2 nad3 nad4L nad5 cob C cox1 R nad1 nad4 atp8 atp6 atp9 cox2 R M nad2b C |
| t4 (3) | rns Y D S N cox3 G nad6 V I S W P rnl T E M M L A F K L Q H M nad2 nad3 atp9 cox2 R nad4L nad5 R cob C cox1 R nad1 nad4 atp8 atp6 |
| | 5 17 18 |
| | rns Y nad6 cox3 K G D S W I S P rnl T E V M M L A F L Q M H cox1 atp9 nad3 cox2 R nad4L nad5 nad2 cob nad1 nad4 R N atp8 atp6 G |
| t5 (4) | rns Y D S N cox3 G nad6 V I S W P rnl T E M M L A F K L Q H M nad2 nad3 atp9 cox2 R nad4L nad5 R cob C cox1 R nad1 nad4 atp8 atp6 |
| | 5 16 17 18 |
| | rns Y nad6 cox3 K G D S W I S P rnl Y E V M M L A F Q L M H cox1 atp9 nad3 cox2 R nad4L nad5 nad2 cob nad1 nad4 R N atp8 atp6 G |
| t6 (1) | rns Y D S N cox3 G nad6 V I S W P rnl T E M M L A F K L Q H M nad2 nad3 atp9 cox2 R nad4L nad5 R cob C cox1 R nad1 nad4 atp8 atp6 |
| | 5 |
| | rns Y nad6 cox3 K G G D S W I S P rnl T E V M M L A F L Q M I H cox1 atp9 A nad3 cox2 R nad4L nad5 nad2 cob C N nad1 I nad4 R N atp8 I atp6 |
| t7 (2) | rns Y D S N cox3 G nad6 V I S W P rnl T E M M L A F K L Q H M nad2 nad3 atp9 cox2 R nad4L nad5 R cob C cox1 R nad1 nad4 atp8 atp6 |
| | 6 17 |
| | rns Y nad6 cox3 atp8 K G D S I W S P rnl T E V M M L A F L Q M H cox1 R R N atp9 cox2 nad4L nad5 nad2 cob nad3 C nad1 nad4 atp6 |
| t8 (2) | rns Y D S N cox3 G nad6 V I S W P rnl T E M M L A F K L Q H M nad2 nad3 atp9 cox2 R nad4L nad5 R cob C cox1 R nad1 nad4 atp8 atp6 |
| | 6 17 |
| | rns Y nad6 cox3 I K G D S W S P rnl T E V M M L A F L Q M H R R N cox1 atp9 cox2 nad4L nad5 nad2 cob nad3 C nad1 nad4 atp8 atp6 |
| t9 (2) | rns Y D S N cox3 nad6 V I S W P rnl T E M M L G A F M K L Q H nad2 nad3 atp9 cox2 R nad4L nad5 R cob C cox1 R nad1 nad4 atp8 atp6 |
| | 14 15 |
| | rns Y D S N cox3 nad6 V I S W P rnl T E M M G L G A F K L Q H M nad2 nad3 atp9 cox2 R R Y nad4L nad5 R cob C cox1 R nad1 nad4 atp8 atp6 |
| t10 (1) | rns Y D S N cox3 nad6 V I S W P rnl T E M M L G A F M K L Q H nad2 nad3 atp9 cox2 R nad4L nad5 R cob C cox1 R nad1 nad4 atp8 atp6 |
| | 4 |
| | rns Y N cox3 K G D S W nad6 V I S P rnl T E M M L A F M L Q H M nad2 nad3 nad4L nad5 cob C cox1 R nad1 nad4 atp8 atp6 atp9 cox2 R M nad2b C |
| t11 (3) | rns Y D S N cox3 nad6 V I S W P rnl T E M M L G A F M K L Q H nad2 nad3 atp9 cox2 R nad4L nad5 R cob C cox1 R nad1 nad4 atp8 atp6 |
| | 9 5 18 |
| | rns Y nad6 cox3 K G D S W I S P rnl T E V M M L A F L Q M H cox1 atp9 nad3 cox2 R nad4L nad5 nad2 cob nad1 nad4 R N atp8 atp6 G |
| t12 (4) | rns Y D S N cox3 nad6 V I S W P rnl T E M M L G A F M K L Q H nad2 nad3 atp9 cox2 R nad4L nad5 R cob C cox1 R nad1 nad4 atp8 atp6 |
| | 9 5 16 18 |
| | rns Y nad6 cox3 K G D S W I S P rnl Y E V M M L A F Q L M H cox1 atp9 nad3 cox2 R nad4L nad5 nad2 cob nad1 nad4 R N atp8 atp6 G |
| t13 (2) | rns Y D S N cox3 nad6 V I S W P rnl T E M M L G A F M K L Q H nad2 nad3 atp9 cox2 R nad4L nad5 R cob C cox1 R nad1 nad4 atp8 atp6 |
| | 9 5 |
| | rns Y nad6 cox3 K G G D S W I S P rnl T E V M M L A F L Q M I H cox1 atp9 A nad3 cox2 R nad4L nad5 nad2 cob C N nad1 I nad4 R N atp8 I atp6 |
| t14 (2) | rns Y D S N cox3 nad6 V I S W P rnl T E M M L G A F M K L Q H nad2 nad3 atp9 cox2 R nad4L nad5 R cob C cox1 R nad1 nad4 atp8 atp6 |
| | 9 6 |
| | rns Y nad6 cox3 atp8 K G D S I W S P rnl T E V M M L A F L Q M H cox1 R R N atp9 cox2 nad4L nad5 nad2 cob nad3 C nad1 nad4 atp6 |
| t15 (2) | rns Y D S N cox3 nad6 V I S W P rnl T E M M L G A F M K L Q H nad2 nad3 atp9 cox2 R nad4L nad5 R cob C cox1 R nad1 nad4 atp8 atp6 |
| | 9 6 |
| | rns Y nad6 cox3 I K G D S W S P rnl T E V M M L A F L Q M H R R N cox1 atp9 cox2 nad4L nad5 nad2 cob nad3 C nad1 nad4 atp8 atp6 |
| t16 (1) | rns Y D S N cox3 nad6 V I S W P rnl T E M M G L G A F K L Q H M nad2 nad3 atp9 cox2 R R Y nad4L nad5 R cob C cox1 R nad1 nad4 atp8 atp6 |
| | 4 |
| | rns Y N cox3 K G D S W nad6 V I S P rnl T E M M L A F M L Q H M nad2 nad3 nad4L nad5 cob C cox1 R nad1 nad4 atp8 atp6 atp9 cox2 R M nad2b C |
| t17 (4) | rns Y D S N cox3 nad6 V I S W P rnl T E M M G L G A F K L Q H M nad2 nad3 atp9 cox2 R R Y nad4L nad5 R cob C cox1 R nad1 nad4 atp8 atp6 |
| | 9 5 17 18 |
| | rns Y nad6 cox3 K G D S W I S P rnl T E V M M L A F L Q M H cox1 atp9 nad3 cox2 R nad4L nad5 nad2 cob nad1 nad4 R N atp8 atp6 G |
| t18 (5) | rns Y D S N cox3 nad6 V I S W P rnl T E M M G L G A F K L Q H M nad2 nad3 atp9 cox2 R R Y nad4L nad5 R cob C cox1 R nad1 nad4 atp8 atp6 |
| | 9 5 16 17 18 |
| | rns Y nad6 cox3 K G D S W I S P rnl Y E V M M L A F Q L M H cox1 atp9 nad3 cox2 R nad4L nad5 nad2 cob nad1 nad4 R N atp8 atp6 G |
| t19 (2) | rns Y D S N cox3 nad6 V I S W P rnl T E M M G L G A F K L Q H M nad2 nad3 atp9 cox2 R R Y nad4L nad5 R cob C cox1 R nad1 nad4 atp8 atp6 |
| | 9 5 |
| | rns Y nad6 cox3 K G G D S W I S P rnl T E V M M L A F L Q M I H cox1 atp9 A nad3 cox2 R nad4L nad5 nad2 cob C N nad1 I nad4 R N atp8 I atp6 |
| t20 (3) | rns Y D S N cox3 nad6 V I S W P rnl T E M M G L G A F K L Q H M nad2 nad3 atp9 cox2 R R Y nad4L nad5 R cob C cox1 R nad1 nad4 atp8 atp6 |
| | 9 6 17 |
| | rns Y nad6 cox3 atp8 K G D S I W S P rnl T E V M M L A F L Q M H cox1 R R N atp9 cox2 nad4L nad5 nad2 cob nad3 C nad1 nad4 atp6 |
| t21 (3) | rns Y D S N cox3 nad6 V I S W P rnl T E M M G L G A F K L Q H M nad2 nad3 atp9 cox2 R R Y nad4L nad5 R cob C cox1 R nad1 nad4 atp8 atp6 |
| | 9 6 17 |
| | rns Y nad6 cox3 I K G D S W S P rnl T E V M M L A F L Q M H R R N cox1 atp9 cox2 nad4L nad5 nad2 cob nad3 C nad1 nad4 atp8 atp6 |

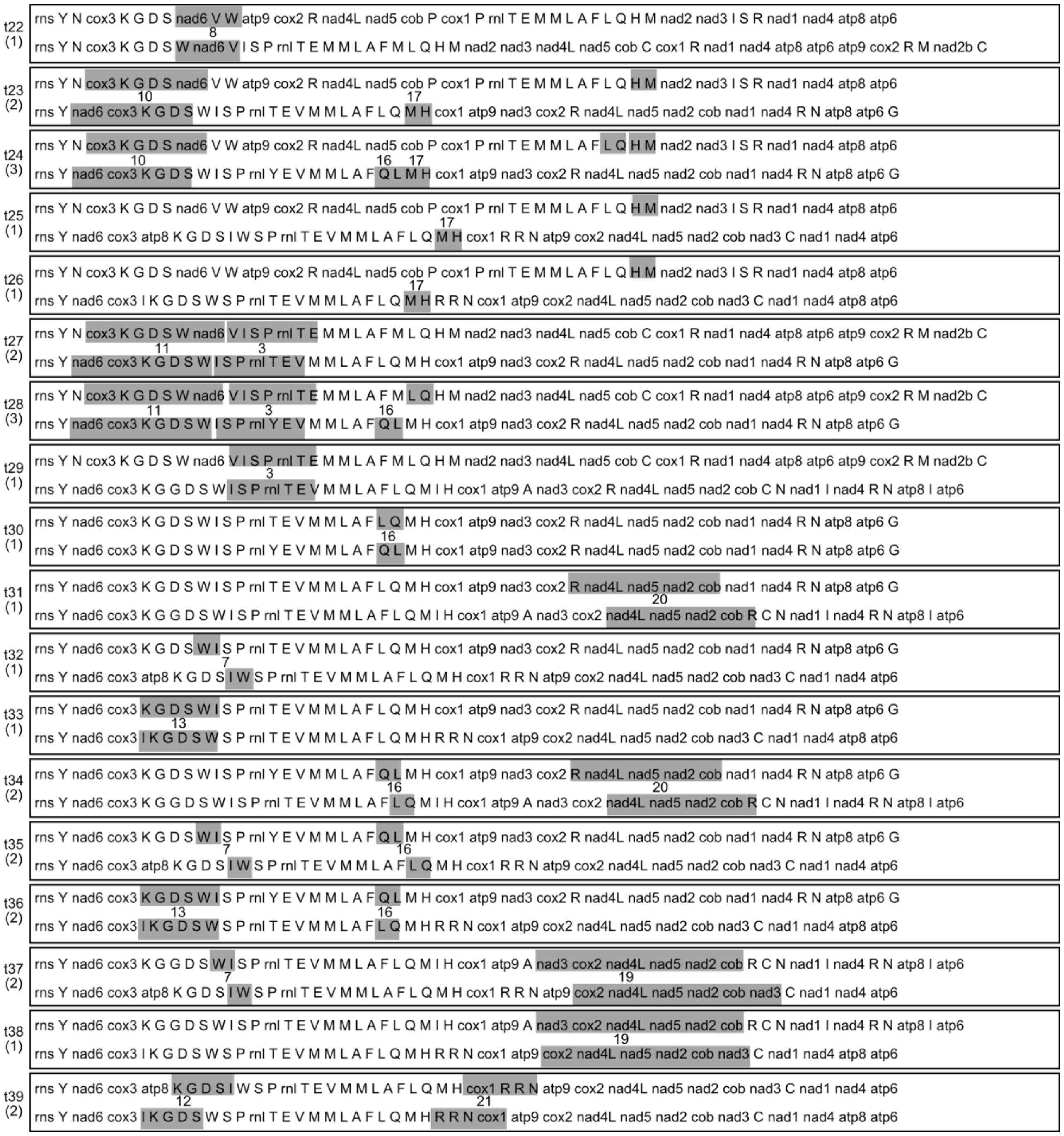

**Figure S4. Putative transposition events in the mitogenomes of 20 fungi detected by the use of CREx algorithm.** Gene orders of *B. bassiana* (BB), *B. pseudobassiana* (BP), *C. brongniartii* (CB), *L. muscarium* (LM), *C. militaris* (CM), *M. anisopliae* (MA), *P. chlarmydosporia* (PC) and *H. jecorina* (HJ) are similar. *F. graminearum* (FG), *F. oxysporum* (FO) and *F. verticillioides* (FV) show similar gene orders, and other two species, *T. rubrum* (TR) and *E. floccosum* (EF), display similar gene order. t1: “BB, BP, CB, LM, CM, MA, PC, HJ” vs *F. solani* (FS); t2: “BB, BP, CB, LM, CM, MA, PC, HJ” vs “FG, FO, FV”; t3: “BB, BP, CB, LM, CM, MA, PC, HJ” vs *N. crassa*

(NC); t4: “BB, BP, CB, LM, CM, MA, PC, HJ” vs *A. niger* (AN); t5: “BB, BP, CB, LM, CM, MA, PC, HJ” vs *A. tubingensis* (AT); t6: “BB, BP, CB, LM, CM, MA, PC, HJ” vs *A. fumigatus* (AF); t7: “BB, BP, CB, LM, CM, MA, PC, HJ” vs “TR, EF”; t8: “BB, BP, CB, LM, CM, MA, PC, HJ” vs *P. brasiliensis* (PB); t9: FS vs “FG, FO, FV”; t10: FS vs NC; t11: FS vs AN; t12: FS vs AT; t13: FS vs AF; t14: FS vs “TR, EF”; t15: FS vs PB; t16: “FG, FO, FV” vs NC; t17: “FG, FO, FV” vs AN; t18: “FG, FO, FV” vs AT; t19: “FG, FO, FV” vs AF; t20: “FG, FO, FV” vs “TR, EF”; t21: “FG, FO, FV” vs PB; t22: VD vs NC; t23: VD vs AN; t24: VD vs AT; t25: VD vs “TR, EF”; t26: VD vs PB; t27: NC vs AN; t28: NC vs AT; t29: NC vs AF; t30: AN vs AT; t31: AN vs AF; t32: AN vs “TR, EF”; t33: AN vs PB; t34: AT vs AF; t35: AT vs “TR, EF”; t36: AT vs PB; t37: AF vs “TR, EF”; t38: AF vs PB; t39: “TR, EF” vs PB. There are 21 transposition events in all, and transpositions were marked, such as two events (1 and 15) in t1: “G nad6 V I S W P rnl T E M M L” vs “nad6 V I S W P rnl T E M M L G”, and “K L Q H M” vs “M K L Q H”.
